# Autoreservoir computing for multistep ahead prediction based on the spatiotemporal information transformation

**DOI:** 10.1038/s41467-020-18381-0

**Published:** 2020-09-11

**Authors:** Pei Chen, Rui Liu, Kazuyuki Aihara, Luonan Chen

**Affiliations:** 1 School of Mathematics, South China University of Technology, Guangzhou, 510640 China; 2 International Research Center for Neurointelligence, The University of Tokyo, Tokyo, 113-0033 Japan; 3 Institute of Industrial Science, The University of Tokyo, Tokyo, 153-8505 Japan; 4 State Key Laboratory of Cell Biology, Shanghai Institute of Biochemistry and Cell Biology, Center for Excellence in Molecular Cell Science, Chinese Academy of Sciences, Shanghai, 200031 China; 5 Key Laboratory of Systems Biology, Hangzhou Institute for Advanced Study, University of Chinese Academy of Sciences, Chinese Academy of Sciences, Hangzhou, 310024 China; 6 Center for Excellence in Animal Evolution and Genetics, Chinese Academy of Sciences, Kunming, 650223 China; 7 Institute of Brain-Intelligence Technology, Zhangjiang Laboratory, Shanghai, 201210 China

**Keywords:** Computational science, Computer science

## Abstract

We develop an auto-reservoir computing framework, Auto-Reservoir Neural Network (ARNN), to efficiently and accurately make multi-step-ahead predictions based on a short-term high-dimensional time series. Different from traditional reservoir computing whose reservoir is an external dynamical system irrelevant to the target system, ARNN directly transforms the observed high-dimensional dynamics as its reservoir, which maps the high-dimensional/spatial data to the future temporal values of a target variable based on our spatiotemporal information (STI) transformation. Thus, the multi-step prediction of the target variable is achieved in an accurate and computationally efficient manner. ARNN is successfully applied to both representative models and real-world datasets, all of which show satisfactory performance in the multi-step-ahead prediction, even when the data are perturbed by noise and when the system is time-varying. Actually, such ARNN transformation equivalently expands the sample size and thus has great potential in practical applications in artificial intelligence and machine learning.

## Abstract (web summary)

Predicting future values of a short time series remains a challenge. Here, the authors propose an auto-reservoir computing framework, which achieved accurate and robust multistep ahead prediction by transforming high-dimensional data into temporal dynamics.

## Introduction

It is a challenging task to make multistep-ahead predictions of a nonlinear dynamical system based only on a short-term time series due to its complicated nonlinearity and insufficient information. Although many methods including statistical regression (e.g., autoregression^1^ and the autoregressive integrated moving average (ARIMA)^2^), machine learning (e.g., the recurrent neural network (RNN)^3^, the long-short-term-memory network (LSTM)^4,5^, support vector regression (SVR)^6,7^, the radial basis function network (RBF)^8^, single-variable embedding (SVE)^9^, and multiview embedding (MVE)^10^), have been applied to the issue of predictability^11–15^, most existing approaches require sufficient training samples or data, e.g., multiple short-time series or a long-term time series, thus failing to reliably predict the future evolution only from a short-term time series. On the other hand, neural networks including the RNN and LSTM can theoretically learn the nonlinear dynamics from the observed data^16–18^. However, these methods typically suffer from the overfitting problem due to the lack of sufficiently many samples when only a single short-term time series is available to train the networks. In addition, training a neural network sometimes costs considerable time and computing resources^19,20^, which also preclude the traditional neural networks from being applied to many real-world systems.

Reservoir computing (RC) is an extension of neural networks recently developed following RNN frameworks that is suitable for temporal/sequential information processing^21^. The role of the reservoir in RC is to nonlinearly transform sequential inputs into a high-dimensional space such that the features of the inputs can be efficiently read out by a simple learning algorithm. Generally, the architecture of RC is feasibly formed by combining two components: a reservoir, which is a hidden neural network of recurrently interconnected nodes (e.g., the RNN itself), and an output or readout layer^22^. RC has drawn much attention because of its dynamical property and easy scalability since the recurrent connections in the network are (e.g., randomly) fixed in advance rather than trained. In other words, training is performed only at the readout stage due to the fixed/random reservoir, thus significantly reducing the training parameters and the computational cost. RC has been applied to a series of real-world studies, such as Great Lakes water level prediction^23^, handwritten digit image recognition^24^, NARMA time-series prediction^25^, limit cycle generation^26^, and temporal parity tasks^27^. However, the current RC framework requires knowledge of the external/additional dynamics that generates the reservoir, which is unrelated to the observed/target system.

Compared with many existing studies on predictions based on long-term time-series data, there have been only a few studies on predictions from short-term but high-dimensional data^28–30^. Nevertheless, the most recent short-term time series usually contains more information on its immediate future evolution than the remote-past time series owing to the time-varying non-stationary nature of many real-world dynamical systems^31–34^. Thus, even if long-term data are measured, prediction effectiveness depends mainly on recent short-term data. On the other hand, notably short-term but high-dimensional data possess rich information due to the high-dimensional variables, whose dynamics is intertwined and thus can be exploited for the prediction. Therefore, it is natural and important to predict future states on the basis of a short-term high-dimensional series, which is also widely available in real-world cases. Actually, by assuming that the steady state is contained in a low-dimensional manifold even for a high-dimensional system that is generally satisfied for dissipative real-world systems, the spatiotemporal information (STI) transformation has theoretically been derived from the delay-embedding theory^35–37^. This method can transform the spatial information of high-dimensional data to the temporal dynamics of any target variable. Based on the STI transformation, the randomly distributed embedding (RDE) framework has been developed for one-step-ahead prediction from the short-term time series by separately constructing multiple maps using the original primary STI equations or the linearized STI equations^36^. Furthermore, the multistep-ahead prediction was also performed by adopting a multilayer neural network as the STI transformation^37^. However, there are two issues that remain unsolved for the prediction: one is the computation cost, and the other is the robustness.

In this study, by taking advantage of both the RC structure and STI transformation, we propose a novel auto-reservoir computing approach, namely, the Auto-Reservoir Neural Network (ARNN), to achieve an accurate, robust and computationally efficient multistep-ahead prediction with short-term high-dimensional data. In particular, we take a nonlinear function *F* as a reservoir structure based on both the primary and conjugate forms of the STI equations, thus constructing the ARNN-based equations. Based on such equations, ARNN encodes *F*(**X**)^*t*^ to **Y**^*t*^ and decodes **Y**^*t*^ to *F*(**X**^*t*^), where **Y**^*t*^ is the temporal (one-dimensional) dynamics across multiple time points and **X**^*t*^ is the spatial (high-dimensional) information at one time point. Hence, ARNN transforms the known spatial information of high-dimensional data to the unknown temporal/dynamical information or prediction of any target variable. On the other hand, the reservoir of ARNN is a multilayer neural network *F* in which the weights among neurons are randomly given and fixed in advance, while the weights of the output are determined directly from solving the ARNN-based STI equations with the dropout scheme^38^. In other words, there is no traditional training process for the neural network in the ARNN algorithm. Unlike traditional RC using external/additional dynamics (irrelevant to the target system), ARNN transforms the dynamics of the observed high-dimensional data as the reservoir, therefore exploiting the intrinsic dynamics of the observed/target system. ARNN actually has a similar form to the autoencoder (i.e., the primary STI for encoding and its conjugate form for decoding), as illustrated in “Methods”. Such a transformation makes ARNN robust.

To validate ARNN, it is applied to a representative model, i.e., a 90-dimensional coupled Lorenz system under different noise and parametric conditions. Furthermore, ARNN is applied to predict many real-world systems. The results show that ARNN achieves multistep-ahead prediction with only a short-term series, which is better than other existing methods in terms of accuracy, efficiency and robustness.

## Results

### ARNN framework with STI transformation

**Fig. 1:**
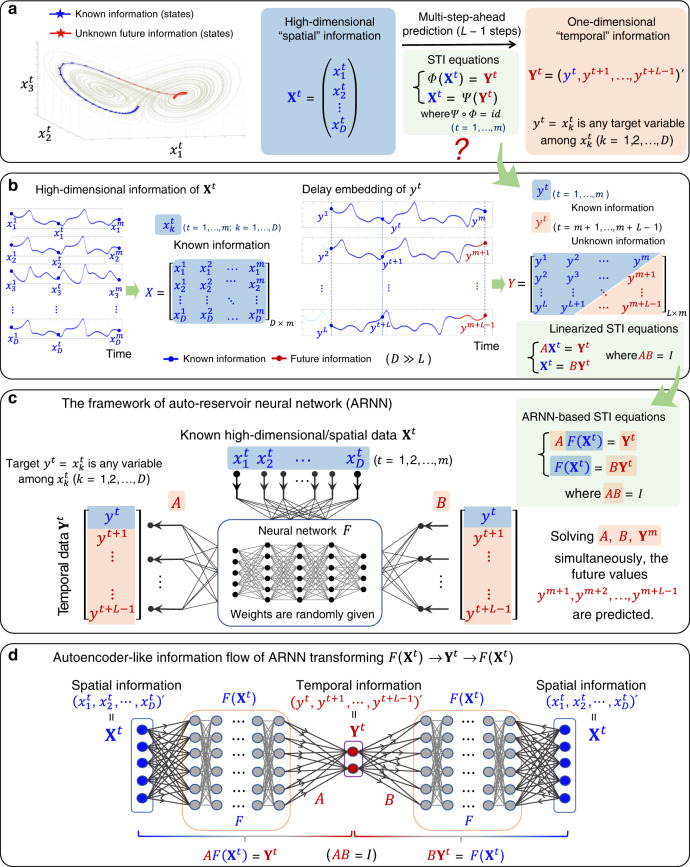
Schematic illustration of the auto-reservoir neural network. **a** Given a short-term time series of a high-dimensional system, it is a challenging task to predict future states of any target variable. For a target variable *y* to be predicted, a delay-embedding strategy is applied, forming a delayed-coordinate vector **Y**^*t*^ corresponding to the observed vector **X**^*t*^ via a function Φ. Such a relation constitutes the spatiotemporal information (STI) transformation with both primary and conjugate forms (STI equations). **b** The linearized STI equations also have primary and conjugate forms. Data can be represented in a matrix form where the future/unknown information {$y^{m + 1},y^{m + 2}, \ldots ,y^{m + L - 1}$} is located in the lower-right triangle of matrix *Y* and the known information {*y*^1^, *y*^2^, …, *y*^*m*^} in the upper-left part of *Y*. **c** Auto-reservoir neural network (ARNN) is a model-free method to make the multistep-ahead prediction for a target *y*. In the ARNN framework, the reservoir component contains a random/fixed multilayer neural network *F*, for which there are time-dependent inputs **X**^*t*^. A target vector **Y**^*t*^ formed by the delay embedding for the prediction is processed through neural network *F* with two weight matrices *A* and *B*. Such an architecture of ARNN is designed to simultaneously solve both primary and conjugate forms of ARNN-based STI equations to enhance the robustness, thus predicting the future information of the target variable *y* even with a short-term time series. **d** According to the information flow, ARNN has an autoencoder-like framework, that is, $F({\bf{X}}^t) \to {\bf{Y}}^t \to F({\bf{X}}^t)$, different from but similar to the autoencoder structure ${\bf{X}}^t \to {\bf{Y}}^t \to {\bf{X}}^t$.

We first describe the primary and conjugate STI equations before constructing ARNN (also see “Methods”). For each observed high-dimensional state ${\mathbf{X}}^t = (x_1^t,x_2^t, \ldots ,x_D^t)^\prime$ with *D* variables and *t* = 1, 2, …, *m*, we construct a corresponding delayed vector **Y**^*t*^ = ($y^t,y^{t + 1}, \ldots ,y^{t + L - 1}$)′ for any target variable *y* to be predicted (e.g., $y^t = x_k^t$) by a delay-embedding strategy with *L* > 1 as the embedding dimension (Fig. 1a), where symbol “′” is the transpose of a vector. Clearly, **X**^*t*^ is a spatial vector with many variables observed at one time point *t* while **Y**^*t*^ is a temporal vector with only one variable *y* but at many time points *t*, *t* + 1, …, *t* + *L* − 1. According to Takens’ embedding theory and its generalized versions, such a delay-embedding scheme **Y**^*t*^ can reconstruct the topologically equivalent dynamics of the original system **X**^*t*^ if *L* > 2*d* > 0 where *d* is the box-counting dimension of the attractor^39–41^. Thus, each spatial vector **X**^*t*^ corresponds to one temporal delayed vector **Y**^*t*^ for each of *t* = 1, 2, …, *m* (Fig. 1a). Thus, the STI equations are

1 $$\left\{ {\begin{array}{*{20}{c}} {{\Phi} \left( {{\mathbf{X}}^t} \right) = {\mathbf{Y}}^t,} \\ {{\mathbf{X}}^t = {\Psi} ({\mathbf{Y}}^t),} \end{array}} \right.$$

where ${\Phi} :{\Bbb R}^D \to {\Bbb R}^L$ and ${\Psi} :{\Bbb R}^L \to {\Bbb R}^D$ are nonlinear differentiable functions satisfying ${\Phi} \circ {\Psi} = id$, symbol “ο” is the function composition operation and *id* represents the identity function (Fig. 1a). In Eq. (1), the first equation is the primary STI equation, and the second is its conjugate form. Note that given *m* observed states **X**^*t*^ (*t* = 1, 2, …, *m*), there are actually *L* − 1 unknown future values of the target variable *y*, i.e., {$y^{m + 1},y^{m + 2}, \ldots ,y^{m + L - 1}$} in **Y**^*t*^ (Fig. 1b). However, it is generally a difficult task to find such a nonlinear function Φ or Ψ. They can be linearized as follows (Fig. 1b) at *t* = 1, 2, …, *m*:

2 $$\left\{ {\begin{array}{*{20}{c}} {A{\mathbf{X}}^t = {\mathbf{Y}}^t,} \\ {{\mathbf{X}}^t = B{\mathbf{Y}}^t,} \end{array}} \right.$$

where *AB* = *I*, *A* and *B* are *L* × *D* and *D* × *L* matrices, respectively, and *I* represents an *L* × *L* identity matrix.

By combining the RC structure and STI transformation, we develop ARNN, which provides multistep-ahead prediction by taking the nonlinear function *F* as a reservoir structure based on both the primary and the conjugate forms of the STI equations (Fig. 1c and Eq. (1)), thus greatly enhancing the prediction robustness, accuracy and computation efficiency. Specifically, a multilayer feedforward neural network *F* is employed for reservoir computing, where the weights among neurons are randomly given in advance. In this study, the neural network contains four layers, with the hyperbolic tangent *tanh* as the activation function, although other appropriate forms of layer designs can also be adopted. Through the processing of the neural network *F*, the original *D* variables ${\mathbf{X}}^t = (x_1^t,x_2^t, \ldots ,x_D^t)^\prime$ are transformed into $\tilde D$ variables $F\left( {{\mathbf{X}}^t} \right) = (F_1({\mathbf{X}}^t),F_2({\mathbf{X}}^t), \ldots ,F_{\tilde D}({\mathbf{X}}^t))^\prime$, where input **X**^*t*^ and output **Y**^*t*^ evolve over time. In other words, the dynamics of the observed high-dimensional data **X**^*t*^ is taken as the reservoir instead of the external/unrelated dynamics as in the traditional RC (see Eq. (7) or (9) in “Methods”), i.e., ARNN can be represented by the following ARNN-based STI equations (Fig. 1c) at *t* = 1, 2, …, *m*:

3 $$\left\{ {\begin{array}{*{20}{c}} {AF\left( {{\mathbf{X}}^t} \right) = {\mathbf{Y}}^t,} \\ {F\left( {{\mathbf{X}}^t} \right) = B{\mathbf{Y}}^t,} \end{array}} \right.$$

where *AB* = *I*, *A* is an $L \times \tilde D$ matrix, *B* is a $\tilde D \times L$ matrix, and *I* represents an *L* × *L* identity matrix. Note that $F_k:{\Bbb R}^D \to {\Bbb R}$ is a nonlinear function (reservoir), $\tilde D$ may be distinct from *D* due to the nonlinear transformation of the neural network *F*, and *A* and *B* are two weight matrices that are determined based on the observed data. Here, the first and second equations in Eq. (3) are the primary and conjugate forms of the STI equations, respectively. Clearly, by solving the ARNN-based STI equations (Eq. (3)) for the given *X* and *F*, we can obtain the future values $\{ y^{m + 1},y^{m + 2}, \ldots ,y^{m + L - 1}\}$ of the target variable as well as the unknown weight matrices *A* and *B*, thus achieving multistep-ahead prediction. Here, *D* > *L* is generally required.

Note that $y^t = x_k^t$ is one variable among all observed variables ${\mathbf{X}}^t = \left( {x_1^t, \ldots ,x_k^t, \ldots ,x_D^t} \right)^\prime$, and ${\mathbf{Y}}^t = \left( {y^t,y^{t + 1}, \ldots ,y^{t + L - 1}} \right)^\prime$. Let ${\mathbf{X}}^t = {\bar{\mathbf{X}}}^t + {\mathbf{I}}_{\mathrm{k}}x_k^t = {\bar{\mathbf{X}}}^t + {\mathbf{I}}_{\mathrm{k}}y^t$, where ${\bar{\mathbf{X}}}^t = (x_1^t, \ldots ,x_{k - 1}^t,0,x_{k + 1}^t, \ldots ,x_D^t)^\prime$ represents all variables except $x_k^t$, and ${\mathbf{I}}_{\mathrm{k}} = (0, \ldots ,0,1,0, \ldots ,0)^\prime$ is a vector where the *k*th position is 1. Then $F\left( {{\mathbf{X}}^t} \right) = f\left( {W^{{\mathrm{in}}}{\bar{\mathbf{X}}}^t + W^{{\mathrm{in}}}{\mathbf{I}}_{\mathrm{k}}{{y}}^t} \right)$, where the function $f = (f_1,f_2, \ldots ,f_{\tilde D})$ represents the elementwise activation functions of the reservoir units. Thus, by noting that **Y**^*t*−1^ includes *y*^*t*^, the first equation of Eq. (3) can be represented as

4 $${\mathbf{Y}}^t = AF\left( {{\mathbf{X}}^t} \right) = Af\left( {W^{{\mathrm{in}}}{\bar{\mathbf{X}}}^t + W^{{\mathrm{in}}}{\mathbf{I}}_{\mathrm{k}}y^t} \right) = W^{{\mathrm{out}}}f\left( {W^{{\mathrm{in}}}{\bar{\mathbf{X}}}^t + W{\mathbf{Y}}^{t - 1}} \right)$$

where *W* represents an appropriate matrix linking *y*^*t*^ and **Y**^*t*−1^, e.g., $W = W^{{\mathrm{in}}}{\mathbf{I}}_{\mathrm{k}}{\mathbf{I}}_2^\prime$. Clearly, Eq. (4) has a similar form to the traditional RC (see Eq. (9) in “Methods”) with *A* = *W*^out^ as the weight matrix in the readout. However, instead of the external/additional dynamics **X**^*t*^ in the traditional RC, ARNN directly uses the inherent dynamics **X**^*t*^ or ${\bar{\mathbf{X}}}^t$ of the original system as reservoir. In ARNN, *W*^in^ is randomly given and fixed, and only *W*^out^ and $\{ y^{m + 1},y^{m + 2}, \ldots ,y^{m + L - 1}\}$ are unknown variables which are to be solved based on the observed **X**^*t*^ (*t* = 1, 2, …, *m*). Note that we can also directly adopt (${\bar{\mathbf{X}}}^t$, **Y**^*t*−1^) as the input of *f* instead of $({\bar{\mathbf{X}}}^t,y^t)$ in the equation above. In the same way, the second equation of Eq. (3) can be represented as another form of RC but with the conjugate matrix *B*.

Interestingly, combining the primary and conjugate equations of Eq. (3) leads to a form similar to that of the autoencoder shown in Fig. 1d and Supplementary Fig. 1. Actually, the matrix *A* with *F* maps/encodes the spatial information to the temporal information in the primary STI equation, whereas the matrix *B* with *F* maps/decodes the encoded temporal information to the original spatial information in the conjugate STI equation (Eq. (3)). It should be noted that there is no separate training process in the ARNN scheme. Actually, the ARNN method makes the training and predicting at the same time by solving the conjugated STI equations Eq. (3). The detailed derivation is presented in the “Methods”, and the ARNN algorithm is given in Supplementary Note 3. Recently, there is much attention on physical reservoir computing in which the external/additional reservoir is implemented by electronic, photonic, spintronic, mechanical, and biological systems and devices^42^. Our method, however, clearly shows that we can use the target complex system itself to form the reservoir instead of physical implementation of the external/additional reservoir.

### Performance of ARNN on Lorenz model

To illustrate the mechanism and the basic idea of the ARNN framework, a 90-dimensional coupled Lorenz model^43^

5 $${\dot{\mathbf{X}}}(t) = G({\mathbf{X}}(t);{\mathbf{P}})$$

was employed to generate synthetic time-course datasets under different noise conditions, where *G*(·) is the nonlinear function set of the Lorenz system with ${\mathbf{X}}(t) = (x_1^t, \ldots ,x_{90}^t)^\prime$ and **P** is a parameter vector. The exact Lorenz system and detailed description are provided in Supplementary Note 6.

#### Noise-free situation

**Fig. 2:**
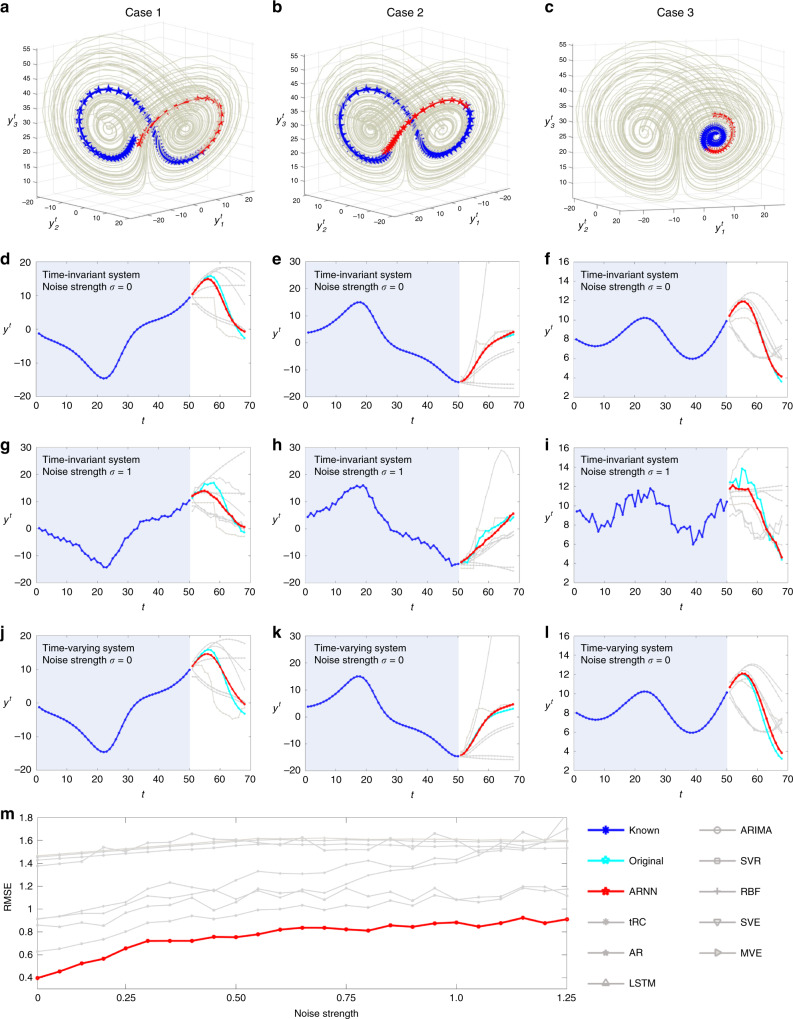
Future state prediction of the Lorenz model based on ARNN. A synthetic time-course dataset was generated in noise-free and noisy situations based on a 90-dimensional coupled Lorenz model. Among the *D* = 90 variables {*x*_1_, *x*_2_, …, *x*_90_}, three targets were randomly selected as *y*_1_, *y*_2_, and *y*_3_. Based on ARNN, future state prediction was carried out for *y*_1_, *y*_2_, and *y*_3_, where the length of the known series/input is *m* = 50, and that of the predicted series is *L* − 1 = 18, i.e., 18-step-ahead prediction. For different initial conditions, there are three cases, where (**a**, **d**) and (**b**, **e**) are the cross-wing cases, i.e., both the known (past) and the unknown (future or to-be-predicted) series are distributed in two wings of the attractor, while (**c**, **f**) is the simpler case, i.e., the known and to-be-predicted series are distributed in a single wing. There are three groups of comparisons for ARNN performance on the original Lorenz system Eq. (5), i.e., the parameters are constants with noise strength *σ* = 0 (**d**–**f**), and noise strength *σ* = 1 (**g**–**i**). For a Lorenz system, Eq. (6) applies with time-varying parameters and noise strength *σ* = 0 (**j**–**l**). With different values of the noise strength, we demonstrated the performance of ARNN and the other methods. The average root-mean-square errors (RMSEs) of 500 cases for ARNN and the other methods are shown in (**m**). The results also demonstrate that ARNN can predict unexperienced dynamics (i.e., in a different wing from the observed data), different from most current deep learning approaches, which generally require a large number of samples to learn all situations.

First, by applying ARNN to a noise-free situation, a series of predictions are presented in Fig. 2, including the cross-wing cases (Fig. 2a, b, d, e), i.e., the known and to-be-predicted series distributed in two wings of the attractor, and the simpler case (Fig. 2c, f), i.e., the known and to-be-predicted series distributed in a single wing of the attractor. For each 3D case (Fig. 2a–c), there are three target variables, *y*_1_, *y*_2_, and *y*_3_, each of which is randomly selected from {*x*_1_, *x*_2_, …, *x*_90_}. In one prediction, we use the 90-dimensional data from the initial 50 steps as known information/input, and ARNN outputs 18-step-ahead prediction for the target variables, i.e., *D* = 90, *m* = 50, and *L* − 1 = 18. For all the noise-free cases (Fig. 2d–f), the predictions achieve high accuracy with root-mean-square errors (RMSEs) of 0.189 in Fig. 2d, 0.0577 in Fig. 2e, and 0.0556 in Fig. 2f, and the predicted dynamical trends for each case agree with the real data. In addition, we present the prediction results for the three targets *y*_1_, *y*_2_, and *y*_3_, respectively, as a 3D prediction in Supplementary Fig. 2. The prediction performance of ARNN from a global perspective of the Lorenz system is provided in Supplementary Fig. 3.

#### Additive noise situation

Second, we discuss the situation when there is additive noise. ARNN (Eq. (3)) and eight traditional prediction methods were applied to the 90D Lorenz system in Eq. (5) with different values of the noise strengths to predict the same target variable. The inputs included data from the former *m* = 50 steps, and the outputs were 18-step-ahead predictions (*L* − 1 = 18). The performance of ARNN and that of the traditional prediction methods were compared. Specifically, three cases are selected in Fig. 2g–i. When noise is added (*σ* = 1), ARNN remains robust and is capable of providing accurate prediction in both future states and dynamical tendency with RMSE = 0.333 in Fig. 2g, 0.292 in Fig. 2h, and 0.277 in Fig. 2i, better than the other methods (RMSE ∈ [0.583, 2.750]). Overall, although the performance slightly deteriorates compared with that of the noise-free situation (Fig. 2d–f), ARNN still captures the dynamics efficiently and is much more robust when the system is perturbed by noise (Fig. 2m), confirming that ARNN works effectively in multistep-ahead prediction based on short-term time series even with noise. The performance of ARNN under different noise conditions is shown in Supplementary Fig. 4.

#### Time-varying system situation

Third, with the same setting (*m* = 50 steps as the input and a prediction *L* − 1 = 18 steps ahead as the output), ARNN was applied to a 90D time-varying/time-switching Lorenz system,

6 $${\dot{\mathbf{X}}}\left( t \right) = G\left( {{\mathbf{X}}\left( t \right);{\mathbf{P}}\left( t \right)} \right),$$

where *G*(·) is the nonlinear function of the Lorenz system with ${\mathbf{X}}(t) = (x_1^t, \ldots ,x_{90}^t)^\prime$; **P**(*t*) is the time-varying/time-switching parameter vector. In other words, the parameters of the Lorenz system change as time evolves; that is, when the time variable *t* moves forward every 10 units, the parameters **P**(*t*) change once. The exact expression of the time-varying Lorenz system and other detailed information are provided in Supplementary Note 6. From Fig. 2j–l, even when the system parameters change over time, ARNN still predicts the future states with high accuracy (RMSE = 0.258 in Fig. 2j, 0.150 in Fig. 2k, and 0.211 in Fig. 2l).

#### Comparison with existing methods

To validate the efficiency of ARNN (Eq. (3)), its short-term prediction performance on the Lorenz system was compared with that of the eight traditional prediction methods, i.e., the traditional reservoir computing (tRC)^25^, AR^1^, LSTM^4,5^, ARIMA^2^, SVR^6,7^, RBF^8^, SVE^9^, and MVE^10^.

In Supplementary Table 1, we summarize the comparisons among ARNN and eight other prediction methods for all 500 predictions of the Lorenz models (Eqs. (5) and (6)). There are three conditions: (i) time invariant and noise free, (ii) time invariant and noise strength *σ* = 1, and (iii) time varying and noise free. Under each condition, the performance of nine prediction methods is compared based on short-term series with parameter sets *m* = 50, *L* − 1 = 18 and *m* = 15, *L* − 1 = 6.

First, for the time-invariant and noise-free cases, when the known length is *m* = 50 and the prediction length is *L* − 1 = 18, the average normalized RMSE of ARNN is 0.397, which is better than those of the other prediction methods with RMSE ∈ [0.608, 1.46]. When the known length becomes even shorter (*m* = 15), the performance of ARNN (RMSE = 0.168) is still better than that of the other methods with RMSE ∈ [0.291, 0.796]; that is, ARNN achieves at least 42% more accuracy than the other methods and is at least 31% faster than the traditional neural network methods. In particular, when the known length is only 15, the LSTM shows a poorer result (RMSE = 0.538) but still incurs three times the running cost of ARNN (Supplementary Table 1). A comparison between ARNN and LSTM on the computational complexity is also demonstrated in Supplementary Note 5.

Second, for the time-invariant and noisy cases with *σ* = 1, ARNN performs better than the other methods; that is, the RMSE of ARNN is 0.884 and RMSE ∈ [1.08, 1.61] for the other methods for the *m* = 50 and *L* − 1 = 18 cases. For the *m* = 15 and *L* − 1 = 6 cases, the RMSE of ARNN is 0.483 and RMSE ∈ [0.678, 1.062] for the other methods, that is, ARNN achieves at least 29% more accuracy than the other methods based on this particular-short-term time series.

Third, for the time-varying and noise-free cases, the RMSE of ARNN is 0.513 and RMSE ∈ [0.863, 2.91] for the other methods for the *m* = 50 and *L* − 1 = 18 cases, and the RMSE of ARNN is 0.284 and RMSE ∈ [0.470, 0.845] for the other methods for the *m* = 15 and *L* − 1 = 6 cases. That is, ARNN achieves at least 40% more accuracy than the other methods.

### The application of ARNN on real-world datasets

In the era of big data, high-dimensional data are ubiquitous in many fields. We apply ARNN to various real-world datasets. The description of the datasets is given in Supplementary Note 6, and the performance of ARNN and the other methods is shown in Table 1.

**Table 1:** Comparison of the performance among ten prediction methods.

Real-world dataset	Metric^a^	Method									
		ARNN	tRC	AR	LSTM	ARIMA	SVR	RBF	SVE	MVE	Linear
Wind speed	RMSE	0.425	3.02	1.13	2.37	1.80	2.48	2.48	2.64	2.08	2.59
	PCC	0.942	−0.268	0.169	−0.124	−0.791	−0.266	−0.301	0.667	0.588	0.315
Solar irradiance	RMSE	0.420	1.74	1.16	1.85	1.18	2.09	2.17	0.612	1.28	0.511
	PCC	0.961	0.318	0.143	0.593	0.582	−0.387	−0.379	0.865	0.614	0.907
Sea-level pressure	RMSE	0.603	3.63	1.12	17.4	1.32	1.43	1.66	1.17	1.29	1.16
	PCC	0.852	−0.295	0.0493	0.0537	−0.215	−0.402	−0.397	0.0453	0.481	0.420
Temperature	RMSE	0.441	5.60	2.78	1.11	2.79	1.49	1.47	1.50	1.51	3.12
	PCC	0.882	0.224	−0.757	0.178	−0.868	−0.102	−0.0364	−0.109	0.458	0.0949
Route of typhoon	RMSE	0.269	6.43	0.565	2.39	0.958	3.49	3.33	1.44	1.56	0.772
	PCC	0.996	0.0499	0.981	−0.147	0.503	−0.745	−0.741	0.503	0.161	0.991
Gene expression	RMSE	0.596	1.65	0.783	1.26	1.47	1.33	1.52	1.25	1.49	1.70
	PCC	0.881	−0.422	0.745	0.0262	−0.217	0.137	0.132	0.343	0.0118	0.213
Stock index	RMSE	0.743	12.2	1.69	50.1	0.787	1.68	1.45	1.07	1.31	1.91
	PCC	0.831	−0.329	−0.0723	−0.353	0.630	−0.176	−0.180	0.225	0.366	0.443
Patient admissions	RMSE	0.551	15.5	2.07	1.56	1.74	2.91	2.95	2.87	2.88	1.96
	PCC	0.814	0.0274	0.0579	0.0676	0.0972	−0.0472	−0.0385	−0.0351	0.308	0.139
Traffic speed	RMSE	1.01	3.08	5.59	11.6	2.38	2.98	7.69	3.64	6.91	14.1
	PCC	0.895	−0.0414	−0.461	−0.211	−0.560	0.154	−0.0792	0.212	−0.371	0.209

^a^The performance metrics include the values of the root-mean-square error (RMSE) and the Pearson correlation coefficient (PCC). The RMSE was normalized by the standard deviation of the real data. The running environment was MATLAB 2019b. The results of the linear method are also summarized in this table.

#### Wind speed prediction

**Fig. 3:**
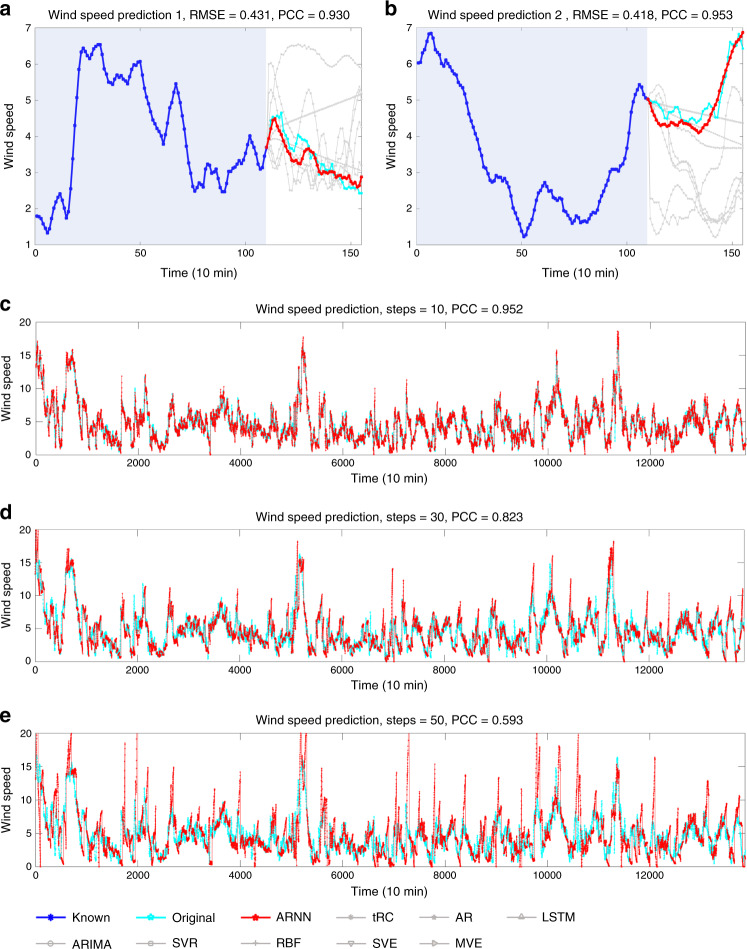
Wind speed prediction in Wakkanai, Japan. Based on the time-course data of *D* = 155 sampling sites in Wakkanai, Japan, ARNN was applied to forecast the wind speed (*m* = 110). The prediction performance of different methods is shown over two periods (*L* − 1 = 45) in (**a**, **b**). The performance of ARNN is significantly better than that of the other methods. The Pearson correlation coefficients (PCCs) between the ARNN prediction result and the original curve are 0.930 (**a**) and 0.953 (**b**). To demonstrate the robustness of our proposed method, ARNN was applied to the whole time series (time point 1–13,860, interval 10 min, 96 days). The results are exhibited for different sets of prediction steps, that is, prediction steps *L* − 1 = 10 (**c**), *L* − 1 = 30 (**d**), and *L* − 1 = 50 (**e**). Clearly, given the fixed known length, predicting a shorter span is more accurate. Overall, the performance of ARNN with different prediction steps is robust and satisfactory for the whole period of 138,600 min.

First, ARNN was applied to a time-course high-dimensional (155-dimensional) dataset of wind speed generated from 155 sampling sites (*D* = 155) in Wakkanai, Japan, provided by the Japan Meteorological Agency^44^. The wind speed was recorded every 10 min during the total period of 138,600 min in 2011. We take the length of the known series as *m* = 110. Based on ARNN, the wind speed of one randomly selected site (i.e., target *y* in Fig. 1) was predicted from 155 sites (i.e., vector **X** of ARNN structure in Fig. 1). The performance of ARNN and the other prediction methods on two segments of time series selected from the total period of 138,600 min are demonstrated in Fig. 3a, b. Specifically, in one-time prediction, ARNN outputs/predicts the wind speed in a future period of 450 min; that is, a prediction of *L* − 1 = 45 steps ahead, while the correlations between the series of predicted points (in red) and that of the real data (in cyan) are all above 0.90 (Fig. 3a, b), although the wind speed is generally considered very difficult to predict. The results predicted by ARNN are better than those by the other methods (Table 1).

To validate the robustness of ARNN toward the multistep-ahead prediction of wind speed, we demonstrate the prediction results for the whole time series (time point 1–13,860 with an interval of 10 min, for a total of 96 days) with different prediction steps (Fig. 3c–e). When the number of prediction steps is *L* − 1 = 50 (Fig. 3e), the overall PCC between the real data of the wind speed and the predicted points is 0.59. The correlation increases to 0.82 and 0.95 if the prediction steps are set to be *L* − 1 = 30 (Fig. 3d) and 10 (Fig. 3c), respectively. The results of the robustness test for different prediction methods are also demonstrated in Supplementary Fig. 7. Clearly, the performance of ARNN with different prediction spans is robust and accurate regardless of the selection of the time region. Thus, this result shows the significant advantage of ARNN in its high robustness because it works well almost everywhere within 138,600 min with various prediction spans, considering that multistep-ahead prediction of wind speed is usually a difficult task.

#### Solar irradiance prediction

**Fig. 4:**
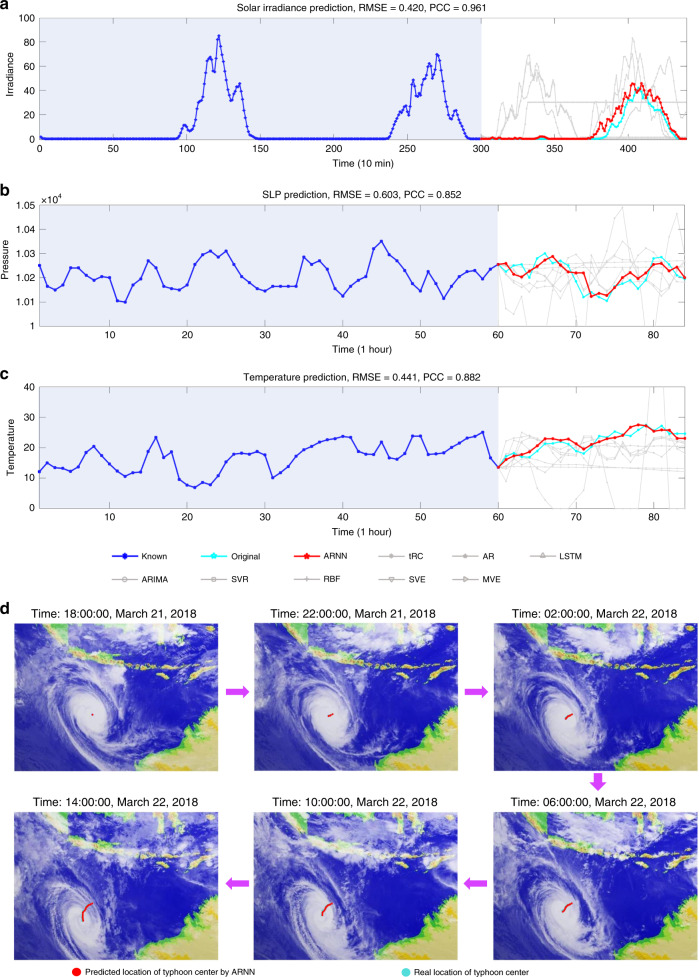
The predicted results of four meteorological datasets. Based on the time-course data of *D* = 155 sampling sites in Wakkanai, Japan, the prediction results are exhibited (**a**). Based on the datasets from Houston, ARNN was applied to forecast (**b**) the sea-level pressure (SLP) and (**c**) the average temperature of the sea. The performance of ARNN is better than that of the other prediction methods. **d** Based on the satellite cloud images of tropical cyclone Marcus (March 2018) collected by the National Institute of Informatics, ARNN predicted the locations of the typhoon center (http://agora.ex.nii.ac.jp/digital-typhoon/). The known information included the initial *m* = 50 images, based on which ARNN outputted *L* − 1 = 21 future locations of the typhoon center.

The second real-world dataset contains solar irradiance data generated from *D* = 155 sampling sites in Wakkanai, Japan. The solar irradiance was recorded every 10 min in 2011. The length of the known series is *m* = 300, i.e., 155-dimensional records from 3000 min (Fig. 4a). Each prediction method outputs/predicts a period of 1400 min (i.e., *L* − 1 = 140 future states). The correlation that reflects the consistency between the real and predicted dynamical trends by ARNN reaches 0.961.

#### Meteorological data prediction

Next, ARNN was applied to the third dataset, a 72-dimensional ground meteorological dataset (*D* = 72) recorded every 1 h, collected from 1998 to 2004 in the Houston, Galveston and Brazoria areas^45^. The sea-level pressure (SLP) and average temperature were predicted as shown in Fig. 4b, c, respectively. For each prediction, the inputs were 72-dimensional data from the former *m* = 60 steps, and the outputs were the 25-step-ahead values of a target index (*L* − 1 = 25).

#### Typhoon Marcus prediction

The fifth dataset, satellite cloud image records of typhoon Marcus, comes from the National Institute of Informatics (http://agora.ex.nii.ac.jp/digital-typhoon/summary/wsp/s/201820.html.en). The dataset is composed of a series of 241 cloud images from 15 March 2018 to 24 March 2018 with one image taken per hour. There are *D* = 2402 variables in each image. Thus, the 241 images can be regarded as a time series within a period of 241 h. For each prediction, the initial *m* = 50 images were regarded as known information, and ARNN was applied to forecast the central position of the tropical cyclone for the next *L* − 1 = 21 time points, i.e., 21-step-ahead prediction in one output. The predicted results are shown in Fig. 4d and Table 1. A movie that shows the dynamical motion route of typhoon Marcus is given in Supplementary Movie 1. The latitudes and longitudes of the central positions are provided in Supplementary Fig. 8.

#### Gene expression prediction in rats

**Fig. 5:**
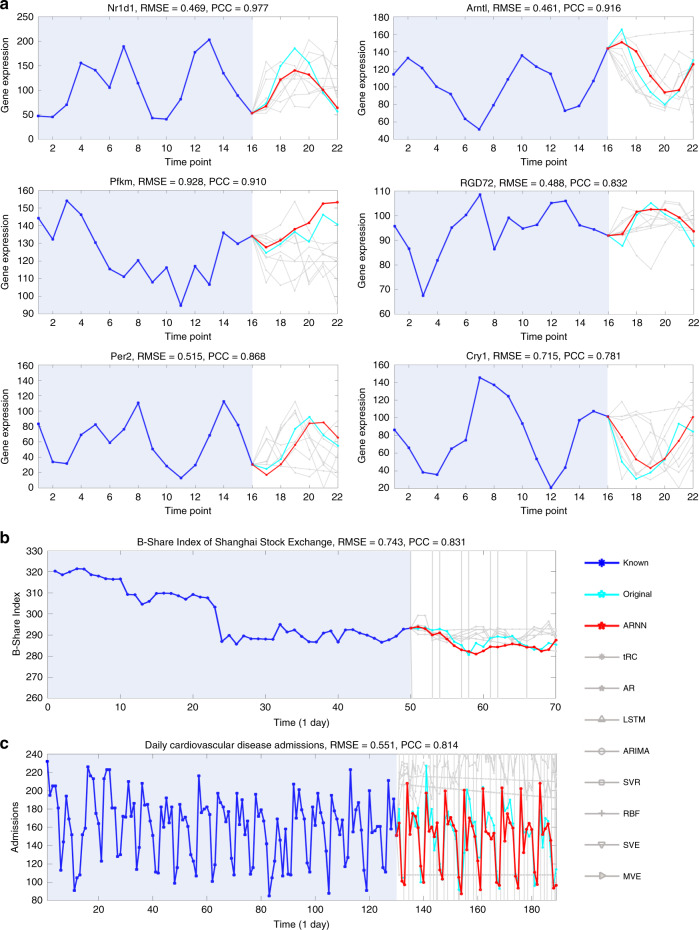
Predictions on gene expressions, the stock index, and patient admissions. **a** Based on the ARNN framework, the dynamical trends of gene expressions in rats were accurately predicted for six circadian rhythm-related genes, i.e., *Nr1d1*, *Arntl*, *Pfkm*, *RGD72*, *Per2*, and *Cry1*. In each prediction, the inputs included the expressions from the initial *m* = 16 time points, and the outputs of the multistep-ahead prediction were the expressions for *L* − 1 = 6 time points ahead. **b** On the basis of *D* = 1130 stock indices of the Shanghai Stock Exchange, the short-term trend of the B-Share Index was predicted, which shows that ARNN achieves relatively high accuracy and strong correlation with the real value. **c** ARNN predicted the dynamical trend of daily cardiovascular disease admissions. The time series ranging from 0 to 130 days were regarded as known information/input, and ARNN predicted the admissions for the *L* − 1 = 60 days ahead. We also compared the ARNN results with the other prediction results for each dataset, which are shown in gray curves. Among the nine prediction methods, the performance of ARNN is the best.

ARNN was then employed to predict the dynamical evolution of gene expressions from a dataset of 84 genes^46^. From the *D* = 84 genes, ARNN was applied to predict the expressions for six target genes, i.e., *Nr1d1*, *Arntl*, *Pfkm*, *RGD72*, *Per2*, and *Cry1* (Fig. 5a). These targets are known as genes related to circadian rhythm, which is a fundamentally important physiological process regarded as the “central clock” of mammals. For each prediction, the known information includes expressions of 84 genes from the initial *m* = 16 time points, and the output is the expressions of the future *L* − 1 = 6 time points, i.e., a 6-step-ahead prediction for the target gene. The predicted expressions by ARNN agree with the observed expressions.

#### Stock index prediction in the Shanghai Stock Exchange

Next, the effectiveness of ARNN was demonstrated in the prediction of a highly unstable system, that is, a high-dimensional stock index dataset from the Shanghai Stock Exchange. This dataset contains the daily (except Saturday and Sunday) values of *D* = 1130 stock indices (variables) from 1 May 2018 to 22 November 2018. Due to the linkage effect in the stock market, different sectors of the stock market interact internally and form a complex system. By applying ARNN with *m* = 50 (days) and *L* − 1 = 20 (days), we predicted the B-Share index of the Shanghai Stock Exchange (Fig. 5b).

#### Cardiovascular inpatient prediction

The prediction accuracy of ARNN was also validated in a real-world dataset that contains several time series, including the index series of air pollutants and the number series of cardiovascular inpatients in major hospitals in Hong Kong^47^. According to the high correlation between the cardiovascular inpatients and air pollutants^48^, ARNN was applied to forecast the inpatient number based on a set of air pollutant indices. Considering the delay effect of every potential factor as well as a dummy vector of the weekday effect^48^, we obtained a 48-dimensional system (*D* = 48). Based on the daily concentrations of pollutants, ARNN predicted the short-term dynamical trend of the daily cardiovascular disease admissions (Fig. 5c), leading to a better result than the other methods (Table 1), where *m* = 130 (days) and *L* − 1 = 60 (days).

#### Traffic speed prediction in Los Angeles

**Fig. 6:**
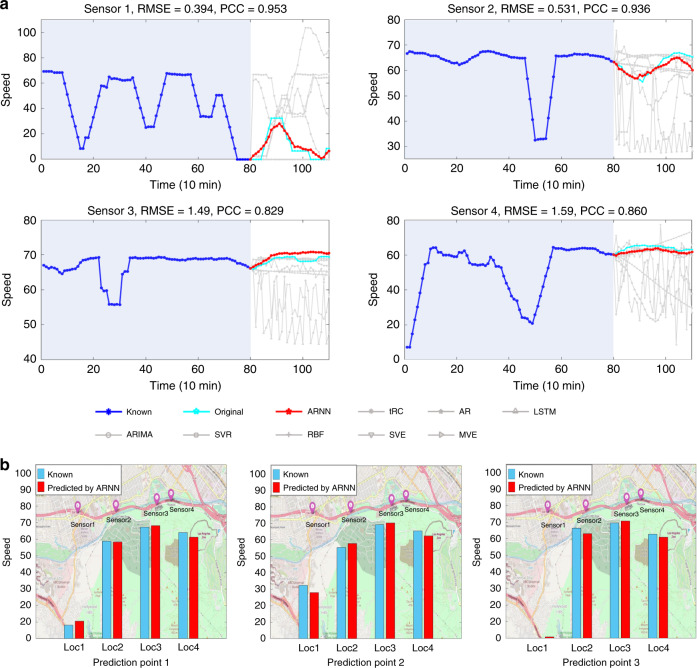
The performance of ARNN prediction on a Los Angeles traffic dataset. **a** The traffic speeds of four nearby locations of Los Angeles (METR-LA) were predicted by ARNN and the other prediction methods. In each prediction, the inputs included the traffic speed (mile/h) from the former *m* = 80 time points, and the outputs were the speeds for *L* − 1 = 30 time points ahead. **b** The results predicted by ARNN for the four nearby locations (Loc1 to Loc4) are shown on the map.

The final application is the prediction of traffic speed (mile/h) based on a dataset collected from *D* = 207 loop detectors in Highway 134 of Los Angeles County^49^. Each detector was regarded as a variable. By applying ARNN, a multistep prediction (*L* − 1 = 30 time points ahead), was obtained from the high-dimensional data with *m* = 80 time points at four adjacent locations (Fig. 6a, b). Supplementary Movie 2 shows the dynamical changes of the predicted and real traffic speeds in four locations. Furthermore, an application of ARNN to the handwriting digits 0–9 from the digit database MNIST is provided in Supplementary Fig. 9, which also illustrates that ARNN is capable of predicting spatial information.

Clearly, these results show that ARNN accurately predicted the dynamical behaviors for non-periodic and highly-fluctuating cases based on only short-term data.

## Discussion

 In this study, we propose the ARNN framework to make multistep-ahead predictions based on short-term high-dimensional data in an accurate, efficient, and robust manner. The delay-embedding theorem ensures that two vectors (the spatial vector **X**^*t*^ and temporal vector **Y**^*t*^) correspond to each other one-by-one via a smooth map^35,36^, and thus we obtain the primary and conjugate STI equations (Eq. (1)), i.e., mapping from **X**^*t*^ to **Y**^*t*^ by Φ and from **Y**^*t*^ to **X**^*t*^ by Ψ. The ARNN method works through transforming the spatial information of high-dimensional variables to the temporal information of a target variable by using both primary and conjugate ARNN-based STI equations (Eq. (3)). Intuitively, as shown in Supplementary Fig. 1, the primary ARNN-based STI equation is an encoder that transforms the spatial information of high-dimensional variables *F*(**X**^*t*^) to the temporal information of a target variable **Y**^*t*^, while the conjugate equation decodes/recovers the (encoded) temporal information **Y**^*t*^ to the high-dimensional variables *F*(**X**^*t*^), i.e., $F({\mathbf{X}}^t) \to {\mathbf{Y}}^t \to F({\mathbf{X}}^t)$, in contrast to the autoencoder ${\mathbf{X}}^t \to {\mathbf{Y}}^t \to {\mathbf{X}}^t$. Solving the conjugated ARNN-based STI equations simultaneously makes the prediction highly robust, as shown in the wind speed prediction for example.

ARNN is computationally efficient and accurate because it incorporates both the STI transformation and the RC structure. On the one hand, by the STI transformation, ARNN transforms the spatial information of high-dimensional data to the temporal information of any target variable, thus equivalently expanding the sample size and alleviating the small sample size problem. On the other hand, by the RC structure, ARNN requires fewer parameters to train, thereby avoiding the overfitting problem. Moreover, in contrast to the external dynamics used in traditional RC, ARNN takes the inherent dynamics of the high-dimensional data themselves as the reservoir.

Notably, most long-term data, such as expression data from biological systems and interest-rate swaps data from financial systems, may also be regarded as short-term data because those systems are generally not stationary but highly time-varying with many hidden variables. Therefore, to characterize or forecast their future states, it is more reliable to employ recent short-term data, than a long-term series of past data. Therefore, ARNN is a general method suitable for many real-world complex systems even when only recent short-term data are available. To check the assumption of STI equations, i.e., the low dimensionality of the underlying attractors, we estimated the box-counting dimensions^50,51^ of all datasets used in this work in Supplementary Table 2, thus validating the low dimensionality of those attractors even though their original dynamics is situated in high-dimensional spaces. Actually, the low dimensionality is relative to the observed high-dimensional variables. In the transient dynamics, the dimension is considered higher than that of the attractor or steady states, but still lower than the number of the observed high-dimensional variables. That is why we can also predict the transient states in many cases in addition to the attractor or steady states (as shown in Table 1 or Figs. 3–6), although the accurate prediction by ARNN on the transient dynamics cannot be theoretically proven. The specific unknown parameters or variables against the known data in each dataset are also summarized in Supplementary Table 3.

One limitation of ARNN is that it is unable to accurately forecast sudden changes or critical transitions in real-world systems. The critical transitions in complex systems are often led by changes in external factors, whose information is generally not included in the measured data^32,52^. On the other hand, the critical transitions resulting from bifurcation can be detected by dynamical network marker methods^53–55^. In addition, ARNN cannot make accurate predictions for strongly noisy data because its theoretical framework is mainly based on deterministic dynamics. Besides, given a low-dimensional sequence, the prediction of ARNN may be similar to the traditional approaches in terms of accuracy due to insufficient spatial information to be transformed.

In summary, compared with traditional prediction methods, ARNN possesses the following advantages. First, ARNN achieves multistep-ahead prediction even with only short-term data due to the transformation from high-dimensional spatial information into temporal information. In contrast to many deep learning methods that suffer from overfitting problems when a large number of parameters are to be trained but with only a single short-time series, ARNN has much fewer parameters due to its reservoir structure where most of the parameters are randomly given. Thus, in practical applications, ARNN requires less computing resources. In particular, ARNN takes the observed high-dimensional variables as the reservoir which represents the inherent dynamics of the target variable, rather than the external dynamics. Third, by simultaneously solving a conjugated pair of STI equations (similar to an autoencoder with both encoding and decoding), ARNN is highly robust and performs well in both noise-perturbed and time-varying-parameter cases, which widely exist in real-world systems. In addition, ARNN has a solid theoretical background based on the delay-embedding theorem. The results for the applications to a variety of real-world problems demonstrate the effectiveness and efficiency of our method. Therefore, ARNN paves a new way for short-term prediction in terms of computationally efficiency, accuracy, and robustness, which is of high potential in real-world applications.

## Methods

The descriptions on the parameters and variables in ARNN framework are summarized in Supplementary Table 4.

### Reservoir computing

Reservoir computing (RC) is a unified computational framework^56,57^, derived from independently proposed RNN models, such as the echo state network (ESN)^58^ and the liquid state machine (LSM)^59^. Generally, ESN is the widely studied RC framework.

ESN uses an RNN-based reservoir consisting of discrete-time artificial neurons^21,58^. When feedback from the output to the reservoir is absent, the time evolution of the neuronal states in the reservoir is described as follows^58^:

7 $${\mathbf{r}}^t = f(W^{{\mathrm{in}}}{\mathbf{X}}^t + W{\mathbf{r}}^{t - 1}),$$

where *t* denotes the discrete time, **r**^*t*^ is the state vector of the reservoir units, **X**^*t*^ is the input vector, *W*^in^ is the weight matrix for the input-reservoir connections, and *W* is the weight matrix for the recurrent connections in the reservoir. The function *f*_*k*_ among *f* = (*f*_1_, *f*_2_, …, *f*_*n*_) represents the *k*th elementwise activation function of the reservoir units, which is typically a sigmoid-type activation function. Equation (7) represents a non-autonomous dynamical system forced by the external input **X**^*t*^. The output is often given by a linear combination of the neuronal states in the reservoir as follows:

8 $${\mathbf{Y}}^t = W^{{\mathrm{out}}}{\mathbf{r}}^t,$$

where **Y**^*t*^ is the output vector and *W*^out^ is the weight matrix in the readout. In supervised learning, this weight matrix is trained to minimize the difference between the network output and the desired output for a certain time period. The performance of ESN depends on the design of the RNN-based reservoir. Here, we consider a special form of RC by combining the neuronal states and output from Eqs. (7) and (8) as

9 $${\mathbf{Y}}^t = W^{{\mathrm{out}}}f(W^{{\mathrm{in}}}{\mathbf{X}}^t + W{\mathbf{Y}}^{t - 1}).$$

In RC, all *W*^in^ and *W* are randomly given and fixed, and only *W*^out^ as unknown variables is trained to minimize the difference between the network output and the desired output, with the known time series (**X**^*t*^, **Y**^*t*^).

### Delay-embedding theorem for dynamical systems

For a general discrete-time dissipative system, the dynamics can be defined as

$${\mathbf{X}}^{t + 1} = \phi \left( {{\mathbf{X}}^t} \right),$$

where $\phi :{\Bbb R}^n \to {\Bbb R}^n$ is a nonlinear map, and its variables are defined in the *n*-dimensional state space ${\mathbf{X}}^t = (x_1^t,x_2^t, \ldots ,x_n^t)^\prime$ at a time point *t* where symbol “′” is the transpose of a vector, and any time interval between two consecutive time points is equal. After a sufficiently long time, all of states are converged into a compact manifold ${\cal{V}}$. The Takens’ embedding theorem is stated as follows^39,40^.

If ${\cal{V}} \subseteq {\Bbb R}^n$ is an attractor with the box-counting dimension *d*, for a smooth diffeomorphism $\phi :{\cal{V}} \to {\cal{V}}$ and a smooth function $h:{\cal{V}} \to {\Bbb R}$, there is a generic property that the mapping ${\Phi} _{\phi ,h}:{\cal{V}} \to {\Bbb R}^L$ is an embedding when *L* > 2*d*, that is,

$${\Phi} _{\phi ,h}\left( X \right) = \left( {h\left( X \right),h \circ \phi \left( X \right), \ldots ,h \circ \phi ^{L - 1}\left( X \right)} \right)^\prime ,$$

where symbol “ο” is the function composition operation. In particular, letting *X* = **X**^*t*^ and *h*(**X**^*t*^) = *y*^*t*^ where $y^t \in {\Bbb R}$, then the mapping above has the following form with ${\Phi} _{\phi ,h} = {\Phi}$ and

$${\Phi} \left( {{\mathbf{X}}^t} \right) = (y^t,y^{t + 1}, \ldots ,y^{t + L - 1})^\prime = {\mathbf{Y}}^t,$$

which is used in our primary STI equations (Eq. (1)). Moreover, since the embedding is one-to-one mapping, we can also derive its conjugate form ${\Psi} :{\Bbb R}^L \to {\Bbb R}^n$ as ${\mathbf{X}}^t = {\Phi} ^{ - 1}({\mathbf{Y}}^t) = {\Psi} ({\mathbf{Y}}^t)$ (Supplementary Note 1). Note that **X**^*t*^ is *n*-dimensional variables here, but sometimes it is used as *D*-dimensional variables (*D* ≤ *n*) in this work.

### STI transformation equations

The steady state or the attractor is generally constrained in a low-dimensional space for a high-dimensional dissipative system, which holds for most real-world complex systems (Supplementary Table 2). By exploring such a low-dimensional feature, spatiotemporal (STI) transformation^35–37^ has theoretically been derived from the delay-embedding theory^39,40^, which can transform the spatial information of high-dimensional data to the temporal information of any target variable. The related description is given in Supplementary Note 2. Assuming *L* > 2*d* where *d* is the box-counting dimension of the attractor, the STI equations (Fig. 1a) can be given as Eq. (1) at *t* = 1, 2, …, *m*, i.e.,

$$\left\{ {\begin{array}{*{20}{c}} {{\Phi} \left( {{\mathbf{X}}^t} \right) = {\mathbf{Y}}^t,} \\ {{\mathbf{X}}^t = {\Psi} ({\mathbf{Y}}^t),} \end{array}} \right.$$

where ${\Phi} :{\Bbb R}^D \to {\Bbb R}^L$ and ${\Psi} :{\Bbb R}^L \to {\Bbb R}^D$ are differentiable functions satisfying ${\Phi} \circ {\Psi} = id$, with symbol “ο” is the function composition operation, and *id* represents the identity function. Clearly, **X**^*t*^ of Eq. (1) is the spatial information of *D* variables while **Y**^*t*^ is the temporal information of the target variable. In Eq. (1), the first equation is the primary form and the second equation is the conjugate form of the STI equations. Intuitively, the primary form encodes the spatial information **X**^*t*^ to the temporal information **Y**^*t*^, while the conjugate form decodes/recovers the encoded temporal information **Y**^*t*^ to the original spatial information **X**^*t*^ (Supplementary Fig. 1).

Based on the STI transformation, the RDE framework has been developed for the one-step-ahead prediction from short-term high-dimensional time-series data^36^, by separately constructing a large number of primary STI transformations. Furthermore, the multistep-ahead prediction is also performed by using a multilayer neural network to represent only the primary STI equation^37^.

The STI equations (Eq. (1)) or one-to-one maps Φ and Ψ hold when the following conditions are satisfied based on the delay-embedding theorem^36,37^ even if the system is high-dimensional and nonlinear.

1. The dynamics of the system is constrained to a low-dimensional attractor in a steady state;
2. All variables used in predictions are from the same system;
3. The stochasticity or noise is sufficiently small;
4. The high-dimensional variables are measurable;
5. The system is time-invariant or stationary during a short-term period.

Actually, all of the above conditions are generally approximately satisfied for a real-world system. The conditions above are sufficient conditions to ensure a one-to-one mapping. In practice, even if the conditions are not fully satisfied, e.g., the system is not in a steady state but in a transient state, ARNN also gives an accurate prediction for many cases, since usually the transient dynamics of a dynamical system is also constrained to a lower dimensional space or manifold.

### Linearized STI equations

Generally, Φ and Ψ are nonlinear functions, which can be linearized as Eq. (2) (Fig. 1b) at $t = 1,2, \ldots ,m$, i.e.,

$$\left\{ {\begin{array}{*{20}{c}} {A{\mathbf{X}}^t = {\mathbf{Y}}^t,} \\ {{\mathbf{X}}^t = B{\mathbf{Y}}^t,} \end{array}} \right.$$

where *AB* = *I*, *A* and *B* are *L* × *D* and *D* × *L* matrices, respectively, and *I* represents an *L* × *L* identity matrix. Clearly, the first and second equations represent the linearized primary and conjugate forms, respectively. We can use the matrix form to represent these equations, i.e., AX = *Y* and *X* = *BY* where *X* and *Y* are defined as

$$Y = \left( {\begin{array}{*{20}{c}} {y^1} & {y^2} & \cdots & {y^m} \\ {y^2} & {y^3} & \cdots & {y^{m + 1}} \\ \vdots & \vdots & \ddots & \vdots \\ {y^L} & {y^{L + 1}} & \cdots & {y^{m + L - 1}} \end{array}} \right)_{L \times m},{\,\,}X = \left( {\begin{array}{*{20}{c}} {\begin{array}{*{20}{c}} {x_1^1} \\ {x_2^1} \end{array}} & {\begin{array}{*{20}{c}} {\begin{array}{*{20}{c}} {x_1^2} \\ {x_2^2} \end{array}} & {\begin{array}{*{20}{c}} \ldots \\ \cdots \end{array}} & {\begin{array}{*{20}{c}} {x_1^m} \\ {x_2^m} \end{array}} \end{array}} \\ {\begin{array}{*{20}{c}} \vdots \\ {x_D^1} \end{array}} & {\begin{array}{*{20}{c}} {\begin{array}{*{20}{c}} \vdots \\ {x_D^2} \end{array}} & {\begin{array}{*{20}{c}} \ddots \\ \ldots \end{array}} & {\begin{array}{*{20}{c}} \vdots \\ {x_D^m} \end{array}} \end{array}} \end{array}} \right)_{D \times m},$$

where the lower-right area of *Y* represents the unknown/future information $\{ y^{m + 1},y^{m + 2}, \ldots ,y^{m + L - 1}\}$. The linearized STI equations provide an approximate way to predict *y* by estimating the maps Φ and Ψ via *A* and *B*, respectively, but the accumulated error drastically increases with the prediction horizon. The prediction performance of the linear method based on the linearized STI equations was provided in Supplementary Figs. 3–5, 7 and 8, and in Table 1 for details.

### ARNN-based STI equations

ARNN can be represented by the ARNN-based STI equations Eq. (3) (Fig. 1c) at $t = 1,2, \ldots ,m$ by lifting Eq. (2) to a neural network form with **X**^*t*^ as an input and **Y**^*t*^ as an output, i.e.,

$$\left\{ {\begin{array}{*{20}{c}} {AF\left( {{\mathbf{X}}^t} \right) = {\mathbf{Y}}^t,} \\ {F\left( {{\mathbf{X}}^t} \right) = B{\mathbf{Y}}^t,} \end{array}} \right.$$

where *AB* = *I, A* is an $L \times \tilde D$ matrix, *B* is a $\tilde D \times L$ matrix, and *I* represents an *L* × *L* identity matrix. Note that $F:{\Bbb R}^D \to {\Bbb R}^{\tilde D}$, is represented by a neural network, whose weights are randomly given and fixed in this work.

Equations above or Eq. (3) can also be represented in a matrix form, i.e., *AF*(*X*) = *Y* and *F*(*X*) = *BY* where $F\left( X \right) = (F\left( {{\mathbf{X}}^1} \right), \ldots ,F\left( {{\mathbf{X}}^m} \right))$. Clearly, by solving the ARNN-based STI equations (Eq. (3)) for given **X**^*t*^ or *F*(**X**^*t*^) with *t* = 1, 2, …, *m*, we can obtain the unknown future values $\{ y^{m + 1},y^{m + 2}, \ldots ,y^{m + L - 1}\}$ of the target variable as well as the unknown weight matrices *A* and *B*.

### Computation of ARNN

The ARNN makes the prediction by simultaneously solving both primary and conjugate ARNN-based STI equations Eq. (3). Although solving any one of the pair equations in Eq. (3) can give the multistep-ahead prediction of the target variable, simultaneously solving both the primary and conjugate equations can provide robust results, different from but similar to the mechanism of the autoencoder (see Fig. 1 and Supplementary Fig. 1). Actually, the information flow of ARNN is $F({\mathbf{X}}^t) \to {\mathbf{Y}}^t \to F({\mathbf{X}}^t)$ (or ${\mathbf{X}}^t \to F\left( {{\mathbf{X}}^t} \right) \to {\mathbf{Y}}^t \to F\left( {{\mathbf{X}}^t} \right) \leftarrow {\mathbf{X}}^t$), in contrast to the autoencoder ${\mathbf{X}}^t \to {\mathbf{Y}}^t \to {\mathbf{X}}^t$. Specifically, for given **X**^*t*^ or *F*(**X**^*t*^), we solve the ARNN-based STI equations Eq. (3), which yields the weight matrices *A* and *B*, and the future information of *y*, i.e., $\{ y^{m + 1},y^{m + 2}, \ldots ,y^{m + L - 1}\}$. There are many ways to solve the conjugated equations Eq. (3), such as the Levenberg–Marquardt method^60,61^. An applicable method for solving ARNN is provided in Supplementary Note 3. After sufficient iterations, the to-be-predicted/future values $\left\{ {y^{m + 1},y^{m + 2}, \ldots ,y^{m + L - 1}} \right\}$ can eventually be determined. The ARNN convergence property of RMSE vs. iteration time is given in Supplementary Fig. 6.

It is clear that the future states including $y^{m + 1},y^{m + 2}, \ldots ,y^{m + L - 1}$ are obtained simultaneously by solving ARNN Eq. (3) with the observed time series of length *m*, which is indeed the (*L* − 1)-step-ahead prediction, rather than the one-step-ahead prediction. It should be noted that when we solve the ARNN equations, {*y*^1^, …, *y*^*m*^} are known but {*y*^*m*+1^, …, *y*^*m*+*L*−1^} are unknown future values of the target variable because of the delay embedding. Therefore, it is similar to a semi-supervised learning process. The detailed algorithm of ARNN is given in Supplementary Note 3.

Although high-dimensional data have rich information, they may also have a noisy effect on the prediction if some of the high-dimensional variables contain little information on the target variable. Thus, choosing relevant variables or eliminating irrelevant variables to the target variable from the high-dimensional data may significantly enhance the performance of ARNN in practical applications. Given a time series of *n*-dimensional variables $(x_1^t,x_2^t, \ldots ,x_n^t)_{t = 1,2, \ldots ,m}^\prime$, by calculating the mutual information between the time series $\left\{ {x_i^1,x_i^2, \ldots ,x_i^m} \right\}_{i = 1,2, \ldots ,n}$ and {*y*^1^, *y*^2^, …, *y*^*m*^}, we select the most correlated variables (e.g., *D* variables among all *n* variables) with the target variable *y*, i.e., variables with the high mutual information with *y*, and obtain the following input vectors and matrix

$${\mathbf{X}}^t = \left( {\begin{array}{*{20}{c}} {x_1^t} \\ {x_2^t} \\ {\begin{array}{*{20}{c}} \vdots \\ {x_D^t} \end{array}} \end{array}} \right)_{t = 1,2, \ldots ,m},\,X = \left( {\begin{array}{*{20}{c}} {x_1^1} & {\begin{array}{*{20}{c}} {x_1^2} & \cdots \end{array}} & {x_1^m} \\ {\begin{array}{*{20}{c}} {x_2^1} \\ \vdots \end{array}} & {\begin{array}{*{20}{c}} {\begin{array}{*{20}{c}} {x_2^2} & \cdots \end{array}} \\ {\begin{array}{*{20}{c}} \vdots & \ddots \end{array}} \end{array}} & {\begin{array}{*{20}{c}} {x_2^m} \\ \vdots \end{array}} \\ {x_D^1} & {\begin{array}{*{20}{c}} {x_D^2} & \cdots \end{array}} & {x_D^m} \end{array}} \right)_{D \times m},$$

where *D* is the number of the selected variables with *n* ≥ *D*, and *m* is the length of the known time series.

Compared with traditional neural network method, ARNN takes much less time and computing resources in decoding the intertwined information among massive variables of a complex system, for the future value prediction of the target variable. A comparison between the ARNN and traditional neural network method on the computational complexity is demonstrated in Supplementary Note 5, from which it is seen that ARNN is cost-effective and requires little computing resources comparing with other neural networks such as LSTM.

## Supplementary information

Supplementary Information

## Data Availability

All data needed to evaluate the conclusions are present in the paper and/or the Supplementary Materials. All data are available at https://github.com/RPcb/ARNN.

## Code availability

The code used in this study is available at https://github.com/RPcb/ARNN.
