# Supplementary material for: Autoreservoir computing for multistep ahead prediction based on the spatiotemporal information transformation
Source: Nat Commun. 2020 Sep 11;11:4568. doi: 10.1038/s41467-020-18381-0 (PMC7486927; doi:10.1038/s41467-020-18381-0)
Supplement: Supplementary file 1 — Supplementary Information [file 41467_2020_18381_MOESM1_ESM.pdf]

Supplementary Information for

Autoreservoir computing for multistep ahead prediction based on the spatiotemporal information transformation

Chen et al.

Contents

Supplementary Figures **2**

Supplementary Figure 1. Structures of the autoencoder and ARNN form. 2
Supplementary Figure 2. The future state prediction of Lorenz model based on ARNN. 3
Supplementary Figure 3. The performance of ARNN and Linear method in a global time region of coupled Lorenz system. 4
Supplementary Figure 4. The performance of ARNN and a linear method under different noise conditions. 5
Supplementary Figure 5. The performance comparison among ARNN and other methods on four cases. . 6
Supplementary Figure 6. The performances of ARNN and LSTM. 7
Supplementary Figure 7. The performance of robustness test of different prediction methods. 8
Supplementary Figure 8. The prediction of Typhoon eye (Latitude, Longitude) based on ARNN and other nine methods. 9
Supplementary Figure 9. The prediction of MNIST digits based on ARNN. 10

Supplementary Tables **11**

Supplementary Table 1. The performances of prediction methods on the 90D coupled Lorenz system ... 11
Supplementary Table 2. The box-counting dimensions of the datasets used in this paper. 12
Supplementary Table 3. The numbers of the unknown parameters and the known data for each dataset 13
Supplementary Table 4. Summary of parameters and variables in ARNN framework 14
Supplementary Movies 14

Supplementary Notes **15**

Supplementary Note 1. Dynamical systems and delay embedding theorem 15
Supplementary Note 2. Spatiotemporal information transformation (STI) equations 15
Supplementary Note 3. ARNN algorithm 16
Supplementary Note 4. The detailed procedure of solving $\boldsymbol{bi} * \boldsymbol{j} *$ 18
Supplementary Note 5. Computational complexity of ARNN 19
Supplementary Note 6. Datasets used in this study 20
Supplementary Note 7. Methods for comparison 23

Supplementary references **26**

Supplementary Figures

Supplementary Figure 1. Structures of the autoencoder and ARNN form.

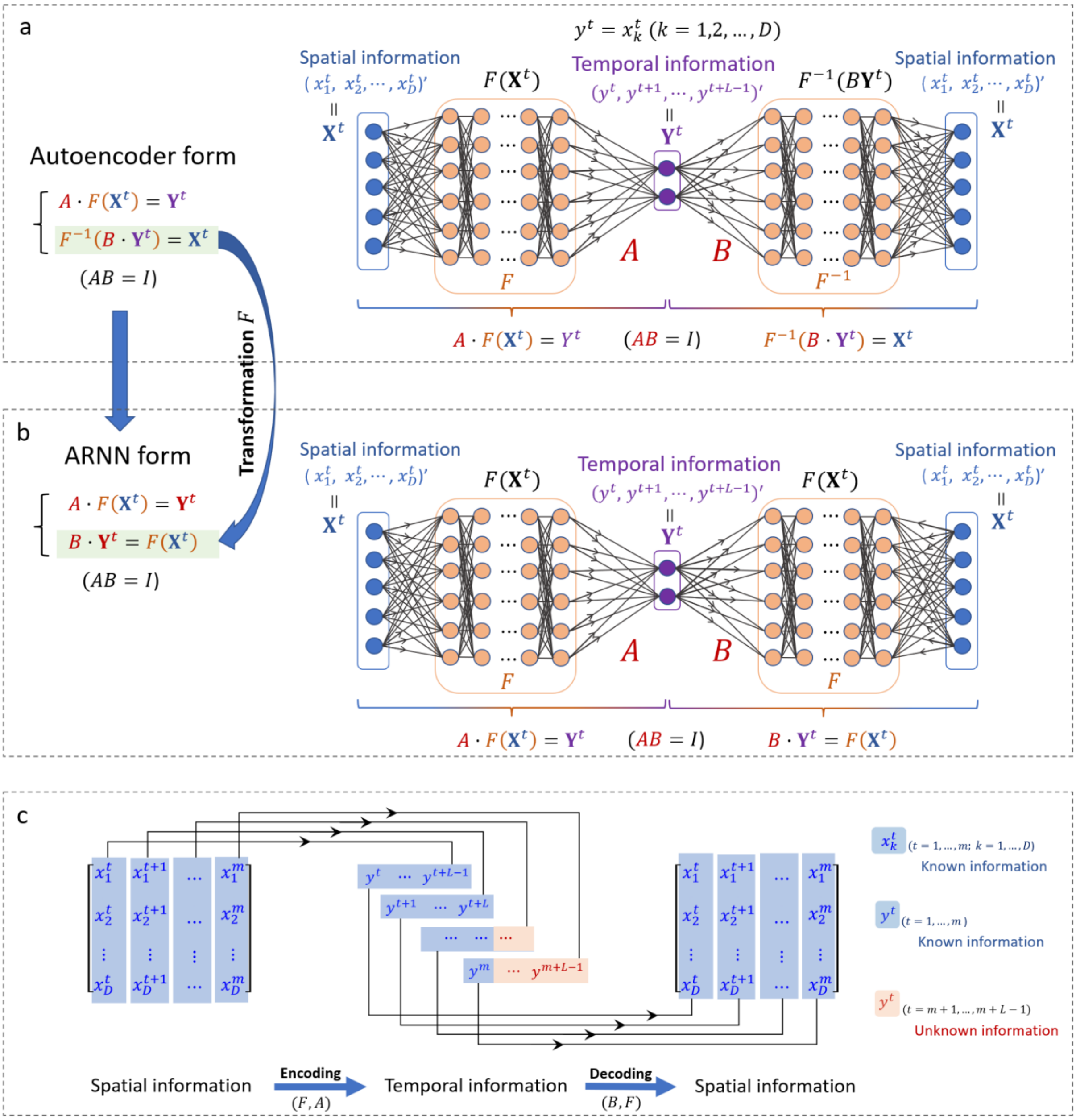

Supplementary Figure 1. Structures of the autoencoder and ARNN form. Information flow of ARNN is $F(\mathbf{X}^t) \to \mathbf{Y}^t \to F(\mathbf{X}^t)$ (or $\mathbf{X}^t \to F(\mathbf{X}^t) \to \mathbf{Y}^t \to F(\mathbf{X}^t) \leftarrow \mathbf{X}^t$), which is different from but similar to autoencoder $\mathbf{X}^t \to \mathbf{Y}^t \to \mathbf{X}^t$. The autoencoder-like form **(a)** can be transformed into the ARNN form **(b)** under the nonlinear transformation F, that is, F acts on both sides of the second equation (the decoder equation) $F^{-1}(B\mathbf{Y}^t) = \mathbf{X}^t$, and then we have $B\mathbf{Y}^t = F(\mathbf{X}^t)$ of ARNN. **(c)** The left-hand-side is to encode the spatial information $\mathbf{X}^t$ to the temporal information $\mathbf{Y}^t$, while the right-hand-side is to decode/recover the encoded temporal information $\mathbf{Y}^t$ to the original spatial information $\mathbf{X}^t$.

Supplementary Figure 2. The future state prediction of Lorenz model based on ARNN.

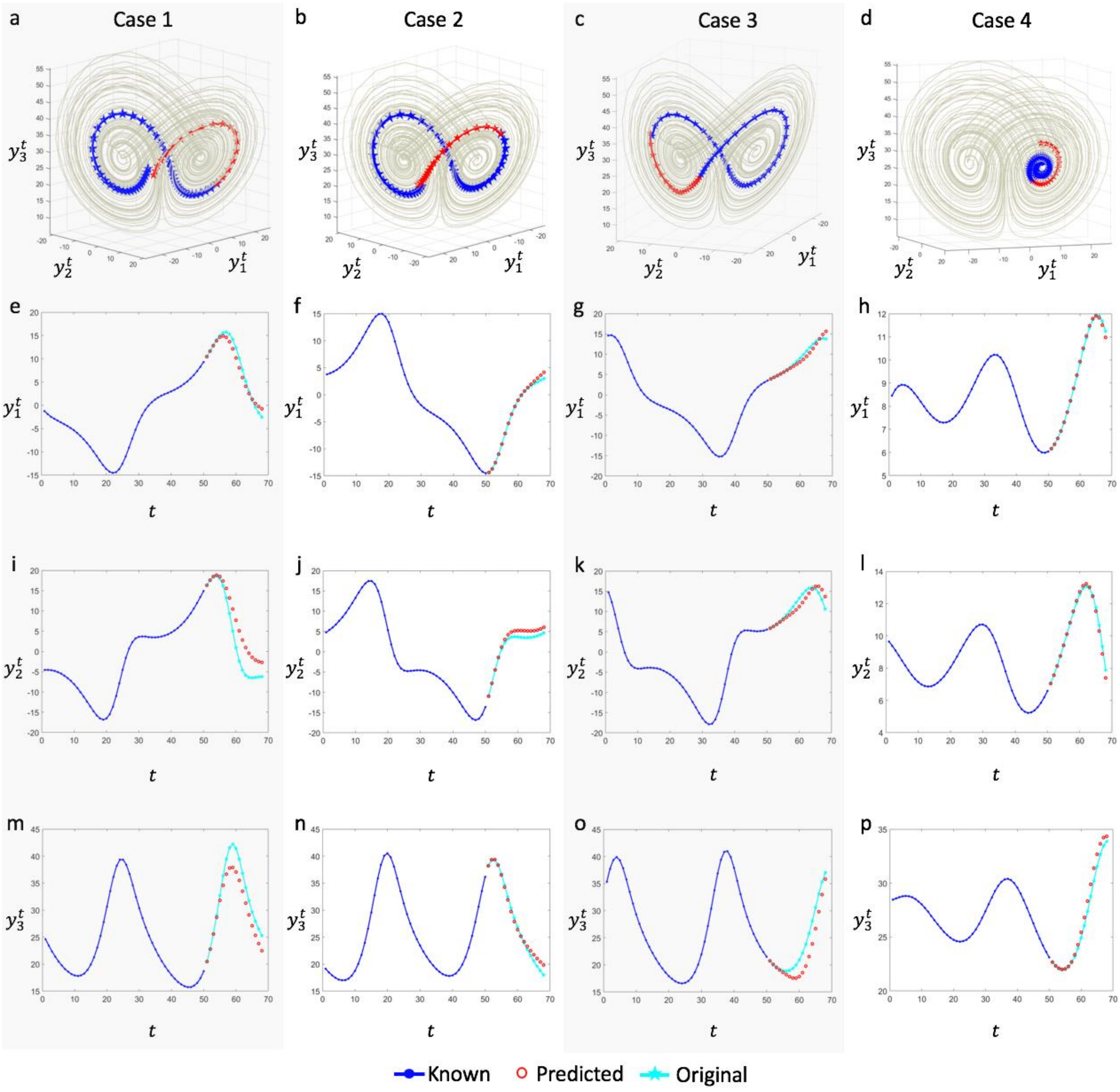

Supplementary Figure 2. The future state prediction of Lorenz model based on ARNN. In the noise-free situation, a synthetic time-course dataset was generated based on the 90-dimensional coupled Lorenz model. Among the $D = 90$ variables $\{x_1, x_2, \dots, x_{90}\}$, three targets were randomly selected as y_1, y_2 and y_3. Through ARNN algorithm, the future state prediction was carried out respectively for y_1, y_2 and y_3, where the length of known series/input is $m = 50$, and that of predicted series is $L - 1 = 18$, i.e., 18-step-ahead prediction in one output. For different initial values, there were four cases, where **(a)**, **(b)**, and **(c)** were the cross-attractors cases, i.e., the known and to-be-predicted series distributed in two attractors, while **(d)** was the periodic case, i.e., the known and to-be-predicted series distributed in a single attractor. For four cases, the blue curves represent the known information. The cyan curves record the real dynamics of the system. The red points are the predicted information based on ARNN. It should be noted that the predicted information (the 18 red points) was obtained in a multi-step-ahead manner, that is, the ARNN provides a period of future information for each single prediction.

Supplementary Figure 3. The performance of ARNN and Linear method in a global time region of coupled Lorenz system.

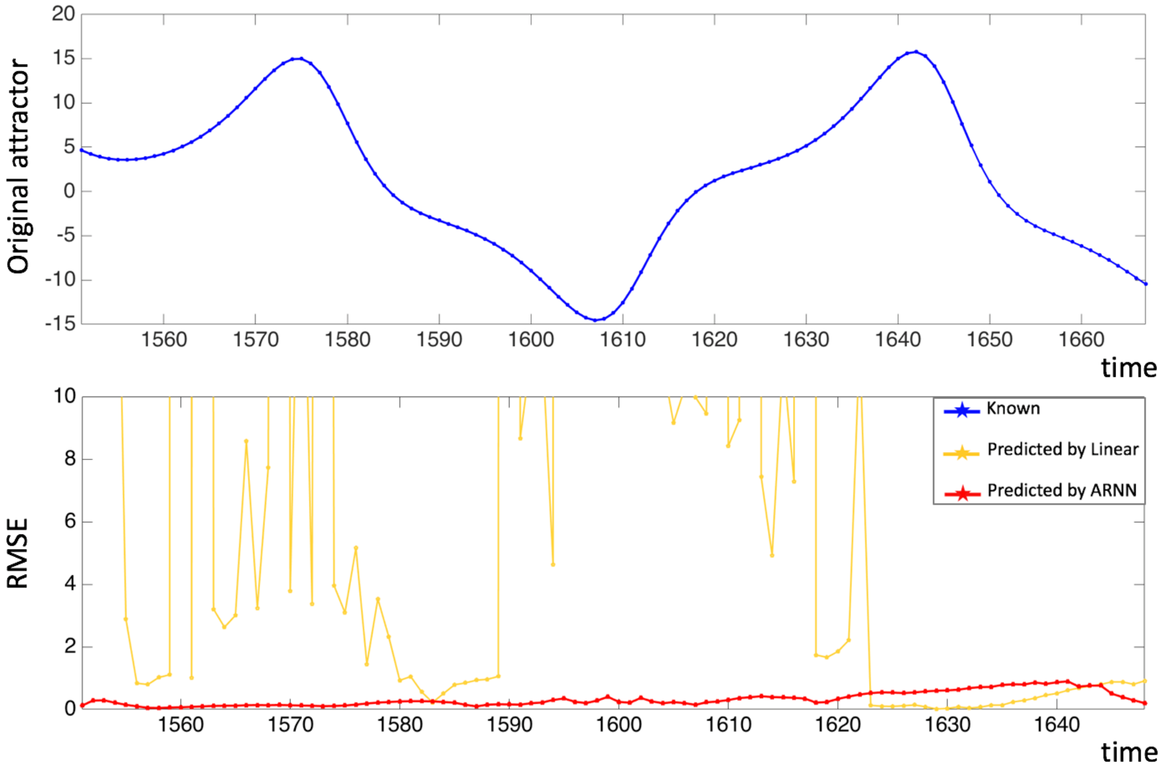

Supplementary Figure 3. The performance of ARNN and Linear method in a global time region of coupled Lorenz system.

Supplementary Figure 4. The performance of ARNN and a linear method under different noise conditions.

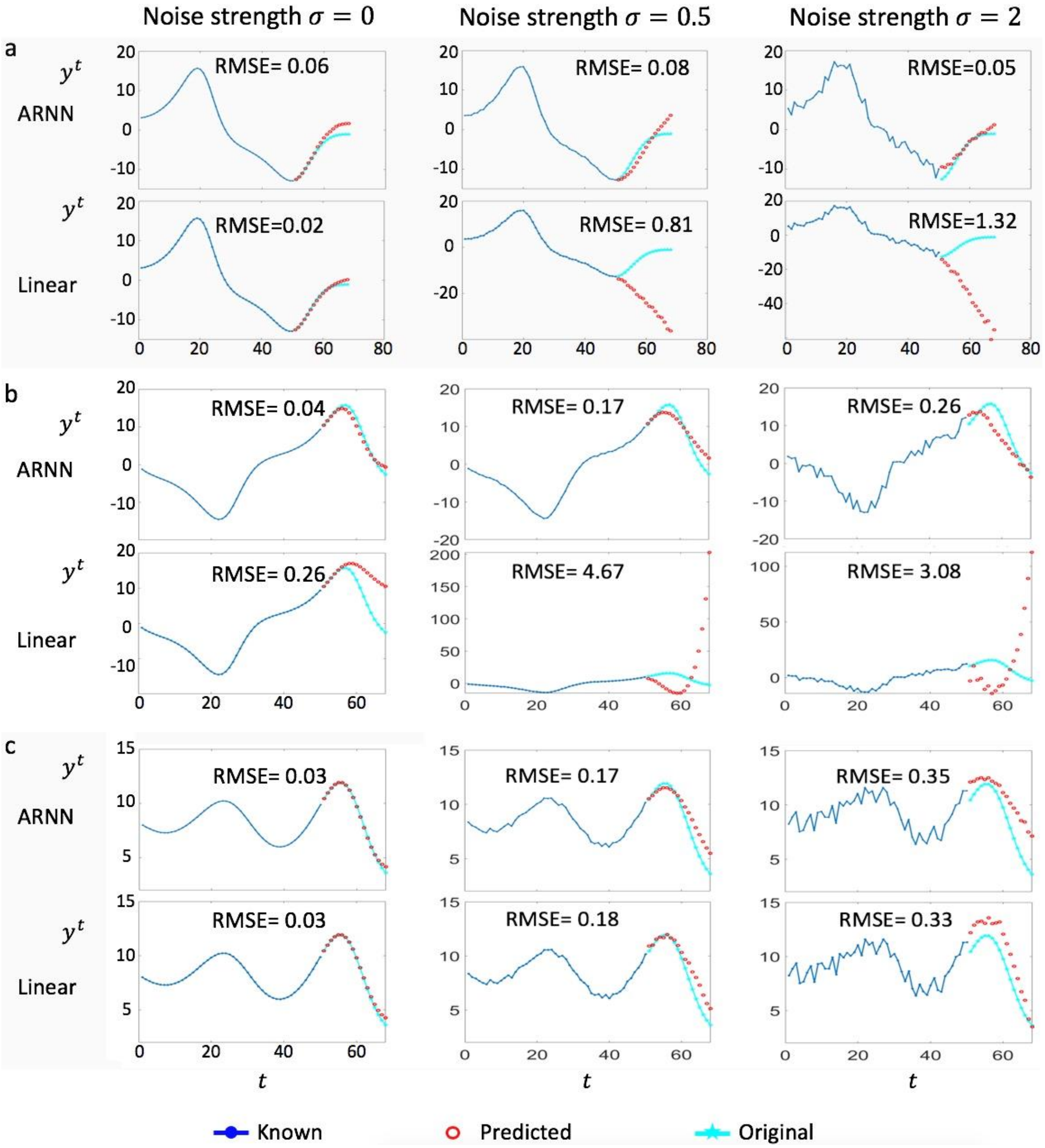

Supplementary Figure 4. The performance of ARNN and a linear method under different noise conditions. With different noise strengths, i.e., $\sigma = 0$, $\sigma = 0.5$, and $\sigma = 2$, we demonstrated the performance of ARNN and the linear method of the linearized STI equations, which is of the same structure of ARNN except the reservoir/nonlinear part. **(a)** The ARNN beats the linear method in the noise-free situation. **(b)** The linear method loses in the noise-free situation. **(c)** Both ARNN and linear method perform well in the noise-free situation. When there is additive noise ($\sigma = 0.5$, or $\sigma = 2$), the performance of ARNN beats that of the linear method for cases (a) and (b).

Supplementary Figure 5. The performance comparison among ARNN and other methods on four cases.

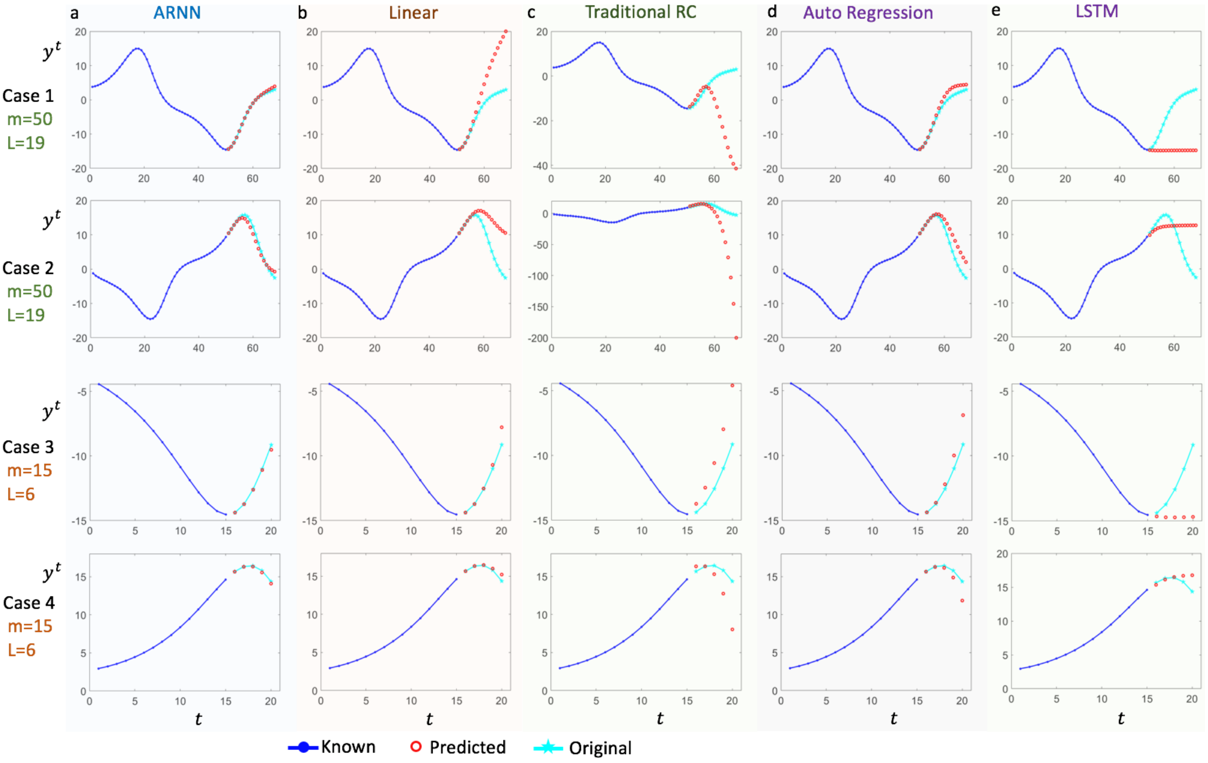

Supplementary Figure 5. The performance comparison among ARNN and other methods on four cases. (a) The performance of ARNN. **(b)** The performance of the linearized STI method (Linear). **(c)** The performance of traditional reservoir computing (tRC). **(d)** The performance of the autoregression (AR). **(e)** The performance of the Long Short-Term Memory network (LSTM).

Supplementary Figure 6. The performances of ARNN and LSTM.

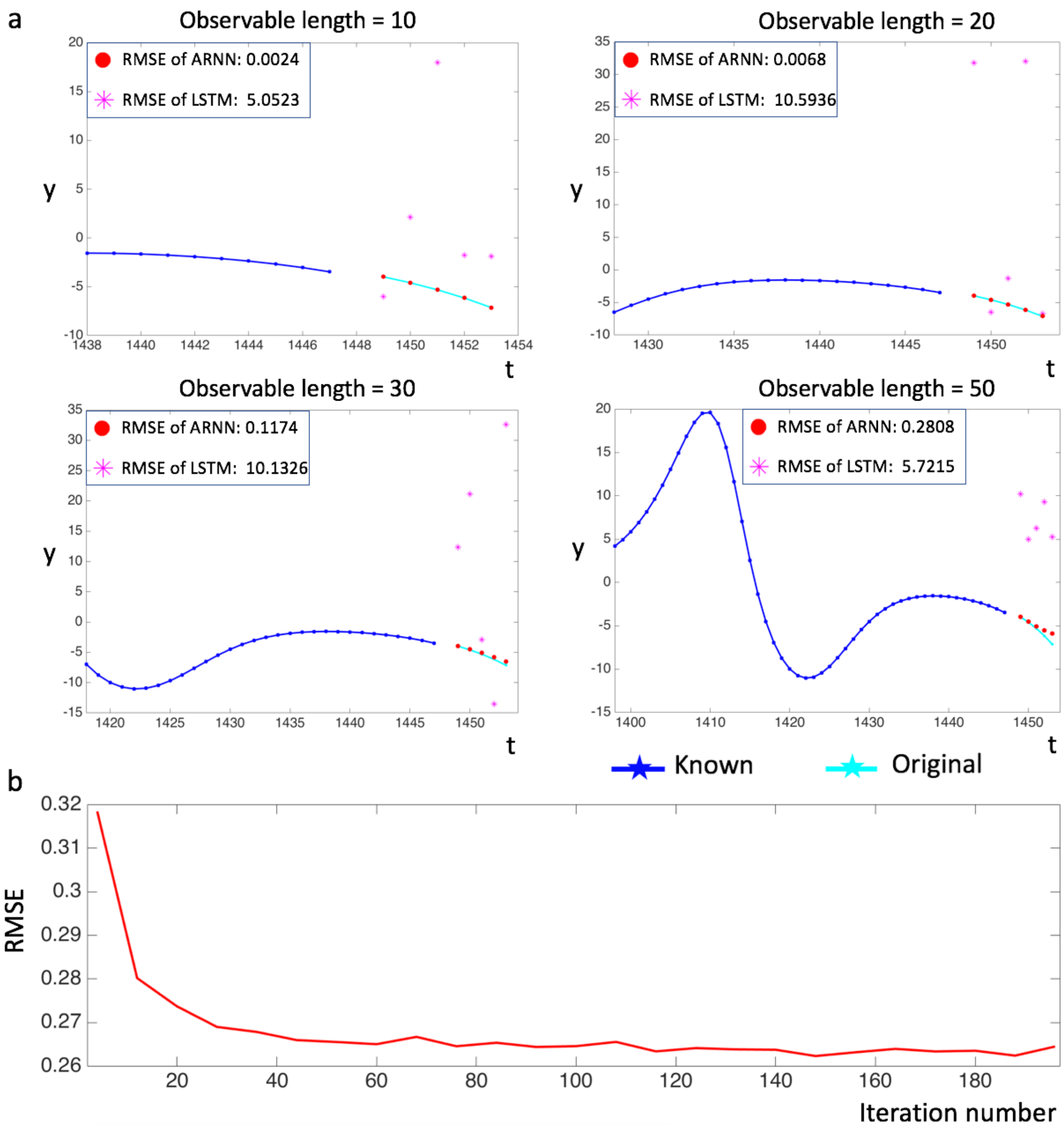

Supplementary Figure 6. The performances of ARNN and LSTM. (a) To compare the performance of ARNN and LSTM, different observable/known length was set to predict a same zone $t \in [1449, 1553]$ of the Lorenz system with time varying parameters, i.e. Eq. (6) in the main text. **(b)** In order to solve the equation set (Eq. (3) in the main text) derived from the ARNN structure, an optimization approach (shown as in Supplementary Notes 3-4) was applied to get A, B and unknown part of y. The curve shows the average RMSE versus iteration number of the optimization method.

Supplementary Figure 7. The performance of robustness test of different prediction methods.

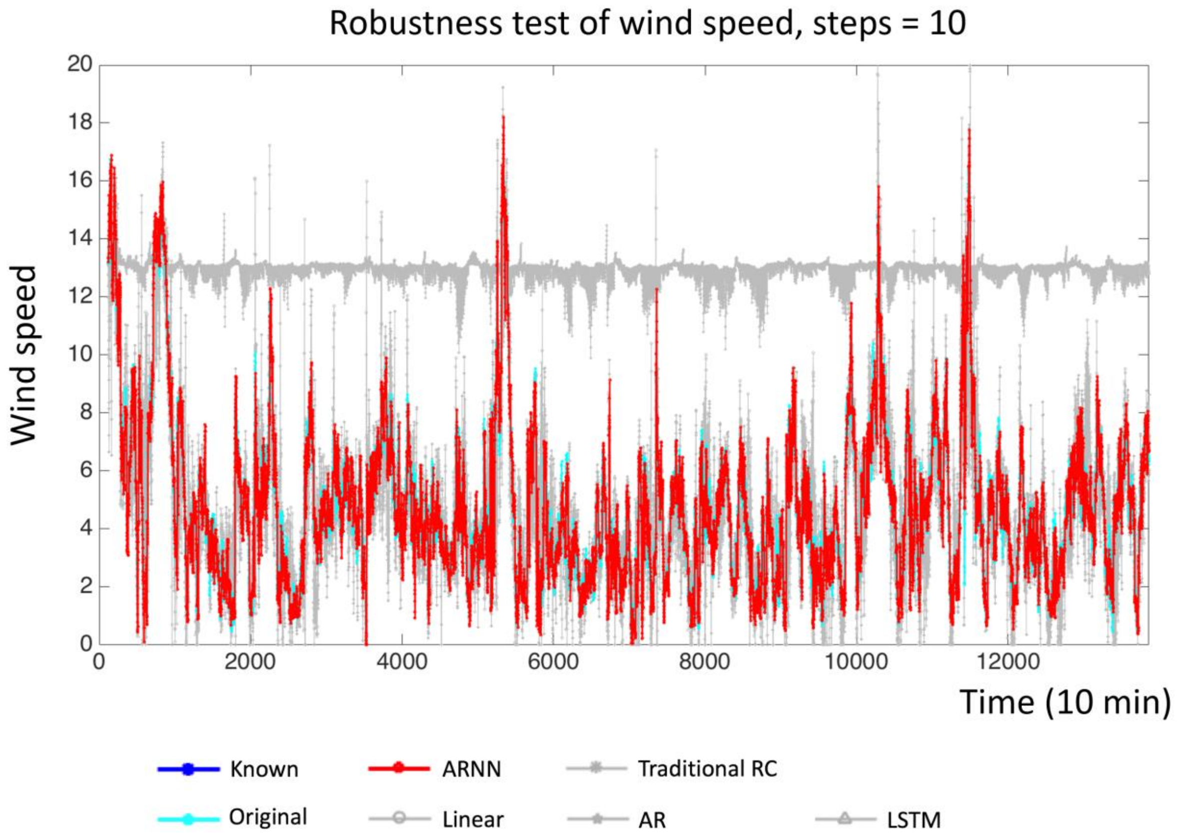

Supplementary Figure 7. The performance of robustness test of different prediction methods. In order to compare the performance of five prediction methods including ARNN, Linear, tRC, AR, and LSTM, the whole time series of wind speed (from 0 to 138,600 minutes) was used to test. The correlations between each predicted series and original series are as follows: $\mathrm{PCC}(\mathrm{ARNN}, \mathrm{Original}) = 0.952$, $\mathrm{PCC}(\mathrm{Linear}, \mathrm{Original}) = 0.759$, $\mathrm{PCC}(\mathrm{tRC}, \mathrm{Original}) = 0.865$, $\mathrm{PCC}(\mathrm{AR}, \mathrm{Original}) = 0.0616$, $\mathrm{PCC}(\mathrm{LSTM}, \mathrm{Original}) = 0.8878$. It is seen that the predicted series via ARNN is the most correlated to the real series.

Supplementary Figure 8. The prediction of Typhoon eye (Latitude, Longitude) based on ARNN and other nine methods.

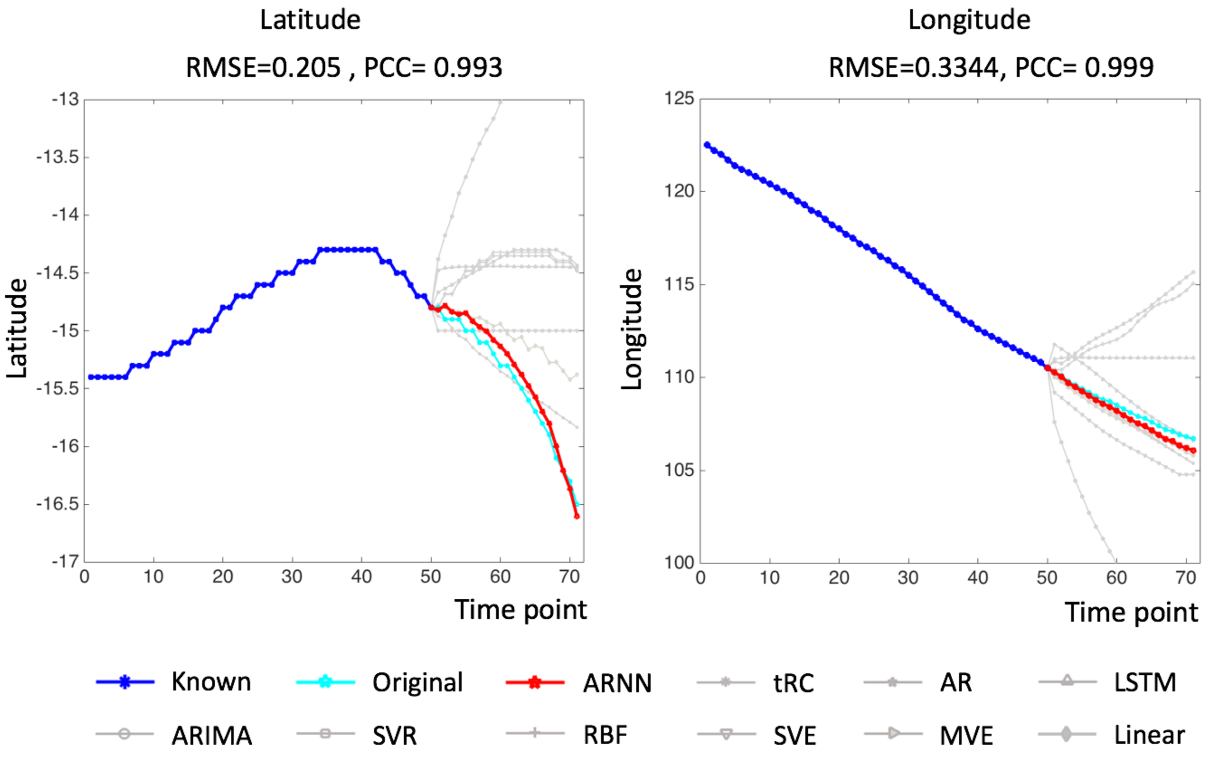

Supplementary Figure 8. The prediction of Typhoon eye (Latitude, Longitude) based on ARNN and other nine methods.

Supplementary Figure 9. The prediction of MNIST digits based on ARNN.

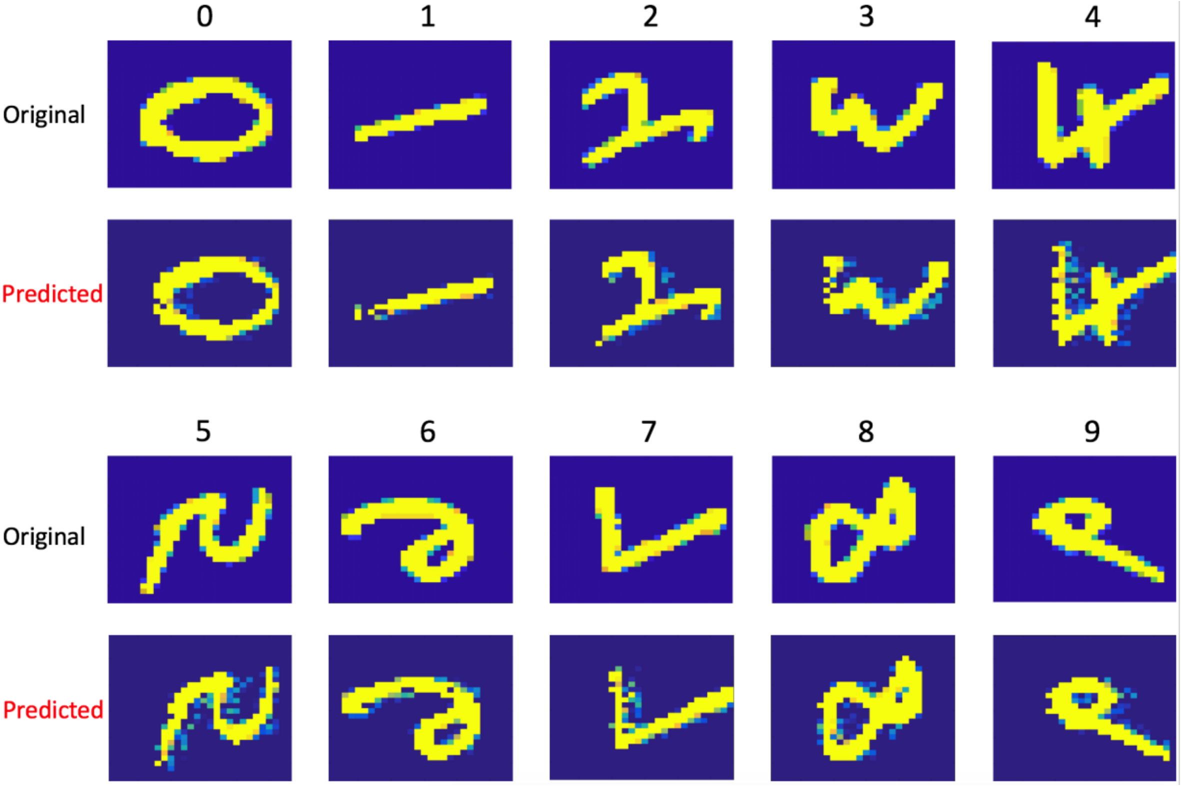

Supplementary Figure 9. The prediction of MNIST digits based on ARNN.

Supplementary Tables

Supplementary Table 1. The performances of prediction methods on the 90D coupled Lorenz system

Data condition		Method / Metric*	**ARNN**	tRC	AR	LSTM	ARIMA	SVR	RBF	SVE	MVE
Noise free, time invariant	*m*=50, *L*-1=18	RMSE	0.397	0.911	0.912	0.861	1.08	1.43	1.46	1.45	0.608
		PCC	0.961	0.855	0.809	0.724	0.804	0.828	0.841	0.833	0.913
		RT	4.33	2.93	0.873	19.1	1.08	3.84	6.33	4.91	15.7
	m=15, *L*-1=6	RMSE	0.168	0.291	0.459	0.538	0.468	0.779	0.761	0.796	0.477
		PCC	0.954	0.914	0.906	0.836	0.873	0.604	0.878	0.877	0.893
		RT	2.27	1.11	0.214	8.91	0.381	2.19	3.31	2.33	12.4
Noise=1, time invariant	*m*=50, *L*-1=18	RMSE	0.884	1.43	1.41	1.09	1.60	1.55	1.61	1.59	1.08
		PCC	0.865	0.755	0.834	0.636	0.786	0.793	0.801	0.699	0.827
		RT	4.76	2.81	0.931	19.4	1.13	3.88	6.26	5.24	17.1
	m=15, *L*-1=6	RMSE	0.483	0.678	0.899	0.937	1.06	0.998	0.956	1.03	0.949
		PCC	0.907	0.845	0.824	0.648	0.724	0.788	0.842	0.672	0.803
		RT	2.33	1.31	0.252	8.75	0.434	2.30	3.62	2.59	13.1
Noise free, time varying	*m*=50, *L*-1=18	RMSE	0.513	2.91	1.21	0.983	1.32	1.48	1.55	1.71	0.863
		PCC	0.924	0.838	0.804	0.719	0.801	0.818	0.822	0.793	0.914
		RT	4.26	2.88	0.812	18.8	1.19	3.65	6.29	5.14	16.6
	m=15, *L*-1=6	RMSE	0.284	0.482	0.470	0.597	0.567	0.823	0.809	0.845	0.520
		PCC	0.934	0.911	0.912	0.854	0.858	0.682	0.877	0.838	0.883
		RT	2.24	1.10	0.209	9.02	0.383	2.21	3.28	2.35	12.6

* For the performance metrics, the values of the root-mean-square error (RMSE), the Pearson correlation coefficient (PCC), and the running time (RT) are the averages from predictions in 500 cases. The RMSE was normalized by the standard deviation of the real data. The RT (CPU time in seconds) was measured on an Intel Xeon E5-2695 v4 2.10 GHz 36-core system with 256 GB RAM. The running environment was MATLAB 2019b.

Supplementary Table 2. The box-counting dimensions of the datasets used in this paper.

Dataset	Estimated dimension*
The 90D coupled Lorenz system	2.68 ± 0.335
Wind speed in Wakkanai, Japan	3.02 ± 0.224
Solar irradiance in Wakkanai, Japan	1.26 ± 0.138
Sea-level pressure & Average temperature in US	2.40 ± 0.206
Route of typhoon center in Indian Ocean	5.64 ± 0.381
Gene expressions related to circadian rhythm	1.86 ± 0.195
B-Share index in Shanghai Stock Exchange	1.92 ± 0.173
Daily number of cardiovascular inpatients	2.65 ± 0.168
Traffic speed in multiple locations in Los Angeles, CA	2.03 ± 0.206

***** The box-counting dimensions are approximately estimated for the data matrix by using the R package "Rdimtools" provided by (Suh, C., You, K. 2018. Rdimtools v. 0.4.2. https://CRAN.R-project.org/package=Rdimtools).

Supplementary Table 3. The numbers of the unknown parameters and the known data for each dataset

Dataset*	Number of known time points ($\boldsymbol{m}$)	Number of total known data ($\boldsymbol{m \times D}$)	To-be-predicted length of unknown y ($\boldsymbol{L-1}$)	Number of unknown elements in A ($\boldsymbol{L \times \tilde{D}}$)	Number of unknown elements in B ($\boldsymbol{\tilde{D} \times L}$)
The 90D coupled Lorenz system	50	50×90	18	19×150	150×19
	15	15×90	6	7×150	150×7
Wind speed in Wakkanai, Japan	110	110×155	45	46×150	150×46
Solar irradiance in Wakkanai, Japan	300	300×155	140	141×150	150×141
Sea-level pressure in the US	60	60×72	25	26×150	150×26
Average temperature in the US	60	60×72	25	26×150	150×26
Route of typhoon center in Indian Ocean	50	50×2402	21	22×150	150×22
Gene expressions related to circadian rhythm	16	16×84	6	7×150	150×7
B-Share index in Shanghai Stock Exchange	50	50×1130	20	21×150	150×21
Daily number of cardiovascular inpatients	130	130×48	60	61×150	150×61
Traffic speed in multiple locations in Los Angeles, CA	80	80×207	30	31×150	150×31

* For each dataset, there are $(L \times \tilde{D}) + (\tilde{D} \times L) + (L-1)$ unknown values against $(m \times D)$ known values in ARNN.

Supplementary Table 4. Summary of parameters and variables in ARNN framework

Symbols	Dimensions	Explanation
Φ	L	Primary STI function
Ψ	D	Conjugate STI function
F	$\widetilde{D}$	A given neural network whose weights are randomly given
$\mathbb{R}^n$	n	n-dimensional real number space
m	1	Scalar, the length of known time series
$L-1$	1	Scalar, the length of to-be-predicted time series for the target variable y
d	1	Scalar, box-counting dimension
t	1	Scalar, time point
n	1	Scalar, the dimension of the observed variables in time-series
D	1	Scalar, the dimension of the (selected) observed variables in time-series
$\widetilde{D}$	1	Scalar, the dimension of F
I	$L \times L$	Matrix, the identity matrix
x_k^t	1	Scalar, the value of the k-th variable in time series at time point t
y^t	1	Scalar, the value of the target variable y at time point t
X	$D \times m$	Matrix, the high-dimensional time series for the observed variables during m time points
$\mathbf{X}^t$	D	Vector, the observed variables at time point t
$\bar{\mathbf{X}}^t$	$D-1$	Vector, the observed variables except y^t at time point t
Y	$L \times m$	Matrix, the delay embedding matrix of y during *m* time points
$\mathbf{Y}^t$	L	Vector, a delay embedding variables of y at time point t
A	$L \times \widetilde{D}$	Matrix, the to-be-solved weights of $F(X^t)$
B	$\widetilde{D} \times L$	Matrix, the to-be-solved weights of Y^t
$\circ$		Function composition operation
$'$		Transpose of a vector
id		The identity function

Supplementary Movies

Movie-Traffic & Movie-Satellite Image are attached to the following link:

https://github.com/RPcb/ARNN

Supplementary Notes

The summary of parameters and variables in ARNN framework is given in Supplementary Table 4.

Supplementary Note 1. Dynamical systems and delay embedding theorem

For a general discrete-time dissipative system, the dynamics can be defined as

$$\mathbf{X}^{t+1} = \phi(\mathbf{X}^t),$$

where $\phi: \mathbb{R}^n \to \mathbb{R}^n$ is a nonlinear map, and its variables are defined in the n-dimensional state space $\mathbf{X}^t = (x_1^t, x_2^t, \dots, x_n^t)'$ at a time point t, where symbol " $'$ " is the transpose of a vector, and any time interval between two consecutive time points is equal. After a sufficiently long time, all of states are converged into a compact manifold $\mathcal{V}$. Denoting $\mathcal{A}$ as the attractor contained in manifold V with the box-counting dimension d, the delay embedding theorem indicates that only using observed long-term data of a single variable can topologically reconstruct the attractor $\mathcal{V}$ of the original high-dimensional system when certain conditions are satisfied. The Takens' embedding theorem is stated as follows[1–3].

If $\mathcal{V}$ is an attractor with the box-counting dimension d, for a smooth diffeomorphism $\phi: \mathcal{V} \to \mathcal{V}$ and a smooth function $h: \mathcal{V} \to \mathbb{R}^1$, there is a generic property that the mapping $\Phi_{\phi,h}: \mathcal{V} \to \mathbb{R}^L$ is an embedding when $L > 2d$, that is,

$$\Phi_{\phi,h}(X) = (h(X), h \circ \phi(X), \dots, h \circ \phi^{L-1}(X))'.$$

where symbol " $\circ$ " is the function composition operation. Generally, the dimension of the original system or the manifold $\mathcal{V}$ is usually much larger than that of attractor $\mathcal{A}$, i.e., $n \gg d$. In particular, letting $X = \mathbf{X}^t$ and $h(\mathbf{X}^t) = y^t$ where $y^t \in \mathbb{R}^1$, then the mapping above has the following form with $\Phi_{\phi,h} = \Phi$ and

$$\Phi(\mathbf{X}^t) = (y^t, y^{t+1}, \dots, y^{t+L-1})' = \mathbf{Y}^t$$

which is used in the following primary STI equations (Supplementary Eq. (1) or main text Eq. (1)). Moreover, since the embedding is one-to-one mapping, we can derive its conjugate form $\Psi: \mathbb{R}^L \to \mathbb{R}^n$ as $\mathbf{X}^t = \Phi^{-1}(\mathbf{Y}^t) = \Psi(\mathbf{Y}^t)$. Note that $\mathbf{X}^t$ is n-dimensional variables here, but sometime it is used as D-dimensional variables ($\leq n$) in this work.

Supplementary Note 2. Spatiotemporal information transformation (STI) equations

The steady-state or the attractor is constrained in a low dimensional space for a high-dimensional system, which also holds for most real-world systems. By exploring such a low-dimensional feature, Spatiotemporal information (STI) transformation[3–5] has theoretically been derived from the delay-embedding theory[2], which can transform

spatial information of high-dimensional data to the temporal information of any target variable. Assuming $L > 2d > 0$ where d is the box-counting dimension of the attractor $\mathcal{A}$ or manifold $\mathcal{V}$, L is the number of embeddings, the STI equations can be stated as Supplementary Eq. (1) or as follows at $t = 1, 2, \dots, m,$ (m is the length of X)

$$\begin{cases} \Phi(\mathbf{X}^t) = \mathbf{Y}^t \\ \mathbf{X}^t = \Psi(\mathbf{Y}^t) \end{cases} \tag{1}$$

where $\Phi: \mathbb{R}^D \to \mathbb{R}^L$ and $\Psi: \mathbb{R}^L \to \mathbb{R}^D$ are differentiable functions satisfying $\Phi \circ \Psi = id$, with symbol " $\circ$ " is the function composition operation, and id represents the identity function. Here, note that X is D dimensions ($\leqslant n$). Clearly, the left-hand side of Supplementary Eq. (1) is the spatial information of D variables while the right-hand side is the temporal information of the target variable. The first equation is the primary form and the second equation is the conjugate form of the STI equations in Supplementary Eq. (1).

Based on STI transformation, randomly distributed embedding (RDE) framework has been developed for one-step-ahead prediction from short-term high-dimensional time-series[4], by separately constructing a large number of partial STI transformations. Furthermore, the multi-step-ahead prediction is also performed by using multi-layer neural network to represent only the primary STI equation[5].

Supplementary Note 3. ARNN algorithm

Given a high-dimensional time series $\mathbf{X}^t = (x_1^t, x_2^t, \dots, x_n^t)'_{t=1,2,\dots,m}$ with length m and dimension n, a to-be-predicted target y is any variable among $x_1, x_2, \dots, x_n$. Based on the STI equations or Supplementary Eq. (1), the linearized STI equations and further ARNN-based STI equations can be derived, and are given in Eq. (2) and Eq. (3) in the Results and Methods of the main text, respectively. The ARNN (auto-reservoir neural network) model is also described in Fig. 1 of the main text and Supplementary Fig. 1. Note that there are many ways to solve the ARNN-based equation (i.e. main text Eq. (3)) based on the observed data $\mathbf{X}^t$ or the given $F(\mathbf{X}^t)$. We design one algorithm in this work. Specifically, as a computational algorithm, ARNN is carried out to uncover the future values $\{y^{m+1}, y^{m+2}, \dots, y^{m+L-1}\}$ of y with the following procedure.

The process of ARNN is iteratively to solve (A,B,Y) of ARNN-based STI equations (Supplementary Eqs. (3)-(5)), respectively, where (A, B) are unknown parameters and

$$Y = \begin{pmatrix} y^1 & y^2 & \cdots & y^m \\ y^2 & y^3 & \cdots & y^{m+1} \\ \vdots & \vdots & \ddots & \vdots \\ y^L & y^{L+1} & \cdots & y^{m+L-1} \end{pmatrix}_{L\times m},$$

which contains the unknown/future values $\{y^{m+1}, y^{m+2}, \dots, y^{m+L-1}\}$ (in the shadow area) of the target variable.

In the detailed procedure, we use the following notations:

- A time series of high-dimensional samples is denoted as $(x_1^t, x_2^t, \dots, x_D^t)'_{t=1,2,\dots,m}$ with D variables and m time points, i.e. the length of known series is m. Symbol " $'$ " is the transpose of a vector.
- The to-be-predicted target $y = x_k$ is any variable among D variables $x_1, x_2, \dots, x_D$.

- L is the predetermined prediction length, i.e. the future values $\{y^{m+1}, y^{m+2}, \ldots, y^{m+L-1}\}$ are to be uncovered.
- Neural network F is randomly given.
- N is the maximum iteration number.

Step 1: Constructing the ARNN-based STI equations from the observed data. Through the feed-forward neural network F, $\mathbf{X}^t$ are transformed into $\widetilde{D}$ variables $F(\mathbf{X}^t) = (F_1(\mathbf{X}^t), \ \ldots, \ F_{\widetilde{D}}(\mathbf{X}^t))'$. Then we have the following ARNN-based STI equations:

$$\begin{cases} A_{L\times\widetilde{D}}[F(\mathbf{X}^1) \quad F(\mathbf{X}^2) \quad \cdots \quad F(\mathbf{X}^m)]_{\widetilde{D}\times m} = Y_{L\times m} \\ B_{\widetilde{D}\times L}Y_{L\times m} = [F(\mathbf{X}^1) \quad F(\mathbf{X}^2) \quad \cdots \quad F(\mathbf{X}^m)]_{\widetilde{D}\times m} \\ A_{L\times\widetilde{D}}B_{\widetilde{D}\times L} = I_{L\times L} \end{cases}, \tag{2}$$

where $I_{L\times L}$ is the identity matrix. It should be noted that, Supplementary Eq. (2) is another form of main text Eq. (3). In Supplementary Eq. (2), all of $\mathbf{X}^t$ or $F(\mathbf{X}^t)$ are available, while the unknowns are $A_{L\times\widetilde{D}}$, $B_{\widetilde{D}\times L}$, and the future values of y, i.e., $\{y^{m+1}, y^{m+2}, \ldots, y^{m+L-1}\}$. In this step, the weights of the neural network or F are randomly given, $A_{L\times\widetilde{D}}$ and $B_{\widetilde{D}\times L}$ are initially given as null matrices (to be updated in Steps 3 and 2 respectively), and $\{y^{m+1}, y^{m+2}, \ldots, y^{m+L-1}\}$ are initialized to be 0.

Step 2: Updating matrix $\boldsymbol{B}$ through a dropout scheme. A part of k ($k < \widetilde{D}$) variables $\tilde{F}(\mathbf{X}^t)$ are randomly chosen from $F(\mathbf{X}^t) = (F_1(\mathbf{X}^t), \ \ldots, \ F_{\widetilde{D}}(\mathbf{X}^t))'$. Solve $\tilde{B}_{k\times L}$ with given $\tilde{A}_{L\times k}$ and $Y_{L\times m}$ by the following equations

$$\begin{cases} \tilde{B}_{k\times L}Y_{L\times m} = [\tilde{F}(\mathbf{X}^1) \quad \tilde{F}(\mathbf{X}^2) \quad \cdots \quad \tilde{F}(\mathbf{X}^m)]_{k\times m} \\ \tilde{A}_{L\times k}\tilde{B}_{k\times L} = I_{L\times L} \end{cases}, \tag{3}$$

where $\tilde{A}_{L\times k}$ is a part of weight matrix $A_{L\times\widetilde{D}}$, while $\tilde{B}_{k\times L}$ is a part of weight matrix $B_{\widetilde{D}\times L}$. Then $B_{\widetilde{D}\times L}$ is updated with the following rules:

(1) If the original element b_{ij} is null, it is replaced directly by the corresponding solution $\tilde{b}_{i^*j^*}$ of Supplementary Eq. (3);

(2) If the element b_{ij} is not null, it is replaced by $\frac{b_{ij}+\tilde{b}_{i^*j^*}}{2}$. Here, b_{ij} is the (i,j)-element of matrix B, $\tilde{b}_{i^*j^*}$ is the (i^*,j^*)-element of matrix $\tilde{B}$. The updating rule is

$$b_{ij}(r+1) = \begin{cases} \tilde{b}_{i^*j^*}, & if\ b_{ij}^r\ is\ null \\ \frac{b_{ij}(r)+\tilde{b}_{i^*j^*}}{2}, & if\ b_{ij}^r\ is\ not\ null \end{cases} \tag{4}$$

where $b_{ij}(r)$ is the value of b_{ij} after r times of updating or iterations with $r = 0, 1, 2, \ldots, N-1$. The procedure of solving $\tilde{b}_{i^*j^*}$ is shown in the below Supplementary Note 4.

Step 3: Updating matrices $\boldsymbol{A}$ and $\boldsymbol{Y}$. Given $B_{\widetilde{D}\times L}$, based on the following Supplementary Eq. (5)

$$\begin{cases} \tilde{A}_{L\times k}[\tilde{F}(\mathbf{X}^1) \quad \tilde{F}(\mathbf{X}^2) \quad \cdots \quad \tilde{F}(\mathbf{X}^m)]_{k\times m} = Y_{L\times m} \\ \tilde{A}_{L\times k}\tilde{B}_{k\times L} = I_{L\times L} \end{cases}, \tag{5}$$

$A_{L\times\tilde{D}} = \left(a_{ij}\right)_{\tilde{D}\times L}$ and the unknown part of $Y_{L\times m}$ are solved as follows:

$$A_{L\times\tilde{D}} \cdot [F(X)|B_{\tilde{D}\times L}] = [Y_{L\times m}|I_{L\times L}], \tag{6}$$

where $[F(X)|B_{\tilde{D}\times L}]$ and $[Y_{L\times m}|I_{L\times L}]$ are augmented matrices.

Step 4: Checking the convergence. The convergence condition of the algorithm is

$$\| \mathbf{Y}_{unknown}(r+1) - \mathbf{Y}_{unknown}(r) \|_{\mathrm{L}} < \varepsilon, \tag{7}$$

where vector $\mathbf{Y}_{unknown} = (y^{m+1}, y^{m+2}, \dots, y^{m+L-1})'$, ε is a small positive number, r is the iteration number, and $\|\cdot\|_{\mathrm{L}}$ is the L^2-norm.

Go to Step 2 for updating matrix $B = \left(b_{ij}\right)_{\tilde{D}\times L}$ in next iteration if the convergence condition is not satisfied. After a sufficiently large number of such iterations, if the convergence condition is satisfied, then matrices $(A_{L\times\tilde{D}}, B_{\tilde{D}\times L})$ as well as the unknown part of $Y_{L\times m}$ are determined and go to Step 5 for output.

Step 5: Output of the future values of $\boldsymbol{y}$. The unknown future values of the target variable y, i.e., $\{y^{m+1}, y^{m+2}, \dots, y^{m+L-1}\}$, are obtained from the converged result of Step 3.

Supplementary Note 4. The detailed procedure of solving $\tilde{\boldsymbol{b}}_{i^*j^*}$

The first equation of Supplementary Eq. (3) is equivalent to the following matrix equation

$$\begin{pmatrix} \tilde{b}_{11} & \tilde{b}_{12} & \cdots & \tilde{b}_{1L} \\ \tilde{b}_{21} & \tilde{b}_{22} & \cdots & \tilde{b}_{2L} \\ \vdots & \vdots & \ddots & \vdots \\ \tilde{b}_{k1} & \tilde{b}_{k2} & \cdots & \tilde{b}_{kL} \end{pmatrix}_{k\times L} \begin{pmatrix} y^1 & y^2 & \cdots & y^m \\ y^2 & y^3 & \cdots & y^{m+1} \\ \vdots & \vdots & \ddots & \vdots \\ y^L & y^{L+1} & \cdots & y^{m+L-1} \end{pmatrix}_{L\times m} = \begin{pmatrix} \tilde{F}_1(\mathbf{X}^1) & \tilde{F}_1(\mathbf{X}^2) & \cdots & \tilde{F}_1(\mathbf{X}^m) \\ \tilde{F}_2(\mathbf{X}^1) & \tilde{F}_2(\mathbf{X}^2) & \cdots & \tilde{F}_2(\mathbf{X}^m) \\ \vdots & \vdots & \ddots & \vdots \\ \tilde{F}_k(\mathbf{X}^1) & \tilde{F}_k(\mathbf{X}^2) & \cdots & \tilde{F}_k(\mathbf{X}^m) \end{pmatrix}_{k\times m}. \tag{8}$$

Supplementary Eq. (8) is expanded as the following equation sets,

$$\begin{cases} \tilde{b}_{s1}y^1 + \tilde{b}_{s2}y^2 + \cdots + \tilde{b}_{sL}y^L = \tilde{F}_s(\mathbf{X}^1) \\ \tilde{b}_{s1}y^2 + \tilde{b}_{s2}y^3 + \cdots + \tilde{b}_{sL}y^{L+1} = \tilde{F}_s(\mathbf{X}^2) \\ \vdots \\ \tilde{b}_{s1}y^{m-L+1} + \tilde{b}_{s2}y^{m-L+2} + \cdots + \tilde{b}_{sL}y^m = \tilde{F}_s(\mathbf{X}^{m-L+1}) \\ \tilde{b}_{s1}y^{m-L+2} + \tilde{b}_{s2}y^{m-L+3} + \cdots + \tilde{b}_{s,L-1}y^m + \tilde{b}_{sL}y^{m+1} = \tilde{F}_s(\mathbf{X}^{m-L+2}) \\ \vdots \\ \tilde{b}_{s1}y^m + \tilde{b}_{s2}y^{m+1} + \cdots + \tilde{b}_{sL}y^{m+L-1} = \tilde{F}_s(\mathbf{X}^m) \end{cases}, s = 1,2,\dots,k. \tag{9}$$

Notice that $\{y^1, y^2, \dots, y^m\}$ are known series. For each s, the first $m-L+1$ equations of Supplementary Eq. (9) contain L unknowns. When $2L-1 \le m$, the number of unknowns is less than or equal to that of equations. Therefore, the elements $\tilde{b}_{i^*j^*}$ can be solved from the first $m-L+1$ equations.

Supplementary Note 5. Computational complexity of ARNN

Compared with the neural network method, ARNN takes much less time and computing resources in decoding the intertwined information among massive variables of a complex system, for the future value prediction of the target variable. The LSTM network, a famous artificial neural network, is widely used in the field of time series analysis, and is thus selected as a representative method for the time cost comparison. Based on multivariate tuning, the LSTM leverages its periodicity for iteration. First, we denote that the size of the training data is N. Because the operation of obtaining the periodic value only goes through one iteration, its time complexity is $O(N)$. Assume that there are n_1 iterations in the iterative tuning part, each of which generates an LSTM network. For each LSTM network, suppose that it has n_2 iterations. Then, a sequence in each iteration has a length of m, and the size of the input data is D. The process of calculating the hidden states of the input data and forward propagation has a time complexity of $m \cdot D$. Then, the adjustment of coefficients according to the Adam algorithm[6] has a time complexity of $m \cdot 1$. Thus, the computational complexity of going through an m-long sequence is $m \cdot (D + 1)$. Lastly, because there are n_2 iterations within which the number of m-long sequences is $\left\lceil \frac{N}{m} \right\rceil$, the computational complexity of training an LSTM network[7] is $O\left(m \cdot (D+1) \cdot \left\lceil \frac{N}{m} \right\rceil \cdot n_2\right) = O(n_2 \cdot N \cdot D)$. In conclusion, for the overall training with n_1 iterations, the total time complexity is $O(N) + n_1 \cdot O(n_2 \cdot N \cdot D) = O(N + n_1 \cdot n_2 \cdot N \cdot D)$. In the multi-step-ahead prediction, since the predicted length is $L - 1$, the final computational complexity of LSTM is $O(L(N + n_1 \cdot n_2 \cdot N \cdot D + s_1^3))$ if there are s_1 neurons in LSTM.

On the other hand, given the weights (W^{in} and W) of the neural network in ARNN, the time complexity of the processing of reservoir converting is $O(s_2^3)$ if there are s_2 neurons in the neural network. A dropout scheme with n iterations is deployed in solving the weight matrix $B_{\widetilde{D}\times L}$. In each iteration, k ($k < \widetilde{D}$) variables are selected, and the time complexity of solving the equations to obtain temporary submatrices of $A_{L\times\widetilde{D}}$, $B_{\widetilde{D}\times L}$ and $Y_{L\times m}$ is $O(2k(\frac{2}{3}L^3 + 2L^2))$, as the computational complexity to solve the indeterminate linear equations is $O(\frac{2}{3}L^3 + 2L^2)$ if there are L coefficients to be solved in the equations. Therefore, the total time cost of n iteration is $O(2kn\left(\frac{2}{3}L^3 + 2L^2\right))$. Finally, the total computational complexity of ARNN is $O(2kn\left(\frac{2}{3}L^3 + 2L^2\right) + s_2^3)$.

For the prediction of short-term data, the length of the training input and that of the predicted sequence are much shorter than the dimension of the input and the total number of iterations, i.e., $L \ll \widetilde{D},\ L \ll D, L \ll n,\ L \ll n_1$, and $L \ll n_2$. Therefore, time complexity (TC) is

$$\mathrm{TC(ARNN)} = \mathrm{O}\left(2kn\left(\frac{2}{3}L^3 + 2L^2\right) + s_2^3\right) \approx O(knL^3 + s_2^3) = O(L \cdot n \cdot k \cdot L \cdot L + s_2^3),$$

$$\mathrm{TC}(LSTM) = O\left((L(N + n_1 \cdot n_2 \cdot N \cdot D) + s_1^3\right) \approx O(Ln_1n_2ND + s_1^3) = O(L \cdot n_1 \cdot D \cdot N \cdot n_2 + s_1^3).$$

Here $n_1, n_2,\ n$ denote the iteration numbers that are of the same TC order in the above algorithms with $D > k$. Additionally, L in ARNN is the step length of the prediction, which is always a short series and much smaller than the training size N in LSTM or iteration number n_2, i.e., $L \ll N$, $L \ll n_2$. In particular, it is generally considered that the time complexity of $O(s_1^3)$ and that of $O(s_2^3)$ are of the same order. Therefore, we obtain

$O(L \cdot n \cdot k \cdot L \cdot L + s_2^3) \ll O(L \cdot n_1 \cdot D \cdot N \cdot n_2 + s_1^3)$, which means that the time complexity of ARNN is smaller than that of LSTM.

Supplementary Note 6. Datasets used in this study

6.1. Coupled Lorenz system

To validate the effectiveness of ARNN in capturing the dynamics of a high-dimensional nonlinear system, we consider a 90D coupled Lorentz system[8]. The *i*th ($i = 1,2,\dots,30$) coupled subsystem is given by

$$\begin{cases} \dot{x}_i = \sigma(t)(y_i - x_i) + Cx_{i-1} \\ \dot{y}_i = \rho x_i - y_i - x_i z_i \\ \dot{z}_i = -\beta z_i + x_i y_i \end{cases}. \quad (10)$$

The coupling term Cx_{i-1} represents that the *i*th subsystem is coupled with the $(i-1)$th subsystem via x component. When $i = 1$, we set $i - 1$ as 30 so that the system could be closed. We set ρ, β and C to be typical values, i.e., $\rho = 28, \beta = 83, C = 0.1$.

It should be noted that in Supplementary Eq. (10) when $\sigma(t) \equiv 10$, Supplementary Eq. (10) is an ordinary Lorenz System (time-invariant system), which was used in Figs. 2a-2i of the main text, Supplementary Figs. 3-5 and Supplementary Table 1. When time-switch parameter $\sigma(t) = 10 + 0.2(t|10)$ is a time-varying parameter with its value $\sigma(t)$ being initially set to be 10 and increased by 0.2 after each ten-time intervals, Supplementary Eq. (10) is a time-switching Lorenz System, which was used in Figs. 2j, 2k and 2l of the main text.

When generating the dataset, we set the initial values of $\{x_i(0), y_i(0), z_i(0)\}_{i=1,2,\dots,30}$ as 0.1 and the time interval Δt as 0.02. Data was collected after transient dynamics. In application, we select different sets of known series, i.e. 15 and 50 points as known data respectively, and then make the predictions (Supplementary Fig. 5 and Supplementary Table 1). Note that ARNN only uses the generated time-course datasets generated from Supplementary Eq. (10) to predict the time evolution as illustration examples, without using Supplementary Eq. (10).

6.2. Wind speed dataset

The wind speed dataset, which is provided by the Japan Meteorological Business Support Center, contains the wind speed (m/s) time series sampled every $\Delta t = 10$ minutes between 2010 and 2012 from $D = 155$ wind stations (variables) in Wakkanai, Japan[9]. As for the 155 stations, their specific locations (latitude and longitude) can be found in the original dataset file 201606241049longitudelatitude.mat accessible in https://github.com/RPcb/ARNN/tree/master/Data/wind%20speed. We use $m = 110$ time points as the known series and make predictions on the next $L - 1 = 45$ time points. As shown in Figs. 3a-3b of the main text, the performance of ARNN is better than the other methods. Besides, utilizing this dataset, we tested the robustness of ARNN with different prediction steps in Figs. 3c-3e of the main text, which proved the effectiveness of ARNN in any time region.

6.3. Solar irradiance dataset

The solar irradiance dataset, which is available via the Japan Meteorological Business Support Center, contains the time series of solar irradiance strength sampled every $\Delta t = 10$ minutes between 2010 and 2012 from $D = 155$ wind stations (variables) in Wakkanai, Japan[9]. We use $m = 300$ time points as the observable data and make predictions on the next $L - 1 = 140$ time points. As shown in Fig. 4a and Table 1, ARNN predicts the solar irradiance.

6.4. Ground meteorology dataset

The ground meteorological dataset contains the one-hour-peak set and can be downloaded in website https://archive.ics.uci.edu/ml/datasets/Ozone+Level+Detection. The data were collected from 1998 to 2004 at the Houston, Galveston and Brazoria area[10]. The dataset contains $D = 72$ variables recorded every 1 hour. Because there are missing values, the mean of neighbor values was employed to fill the missing values. We use $m = 60$ time points as known series and make predictions on the next $L - 1 = 25$ time points. As shown in Figs. 4b-4c, ARNN predicts the trends accurately (Table 1 of the main text).

6.5. Satellite cloud image dataset

This dataset contains satellite cloud images collected by National Institute of Informatics and we select the typhoon Marcus to predict (http://agora.ex.nii.ac.jp/digital-typhoon, 2019). The dataset is composed of a series of 241 cloud images ($D = 2402$ variables) from 2018-3-15 to 2018-3-24 with one image taken per hour. The known series was set as $m = 50$ time points/images. Then ARNN predicted the typhoon center for the next $L - 1 = 21$ time points. As shown in Table 1 and Fig. 4d, ARNN predicts the route of typhoon center accurately. The exact positions of the predicted typhoon center, i.e., the latitude and longitude of central position, were provided in Supplementary Fig. 8. A movie of the dynamical change of the typhoon center is provided in the following link: https://github.com/RPcb/ARNN

6.6. Gene expression dataset of rats

This dataset is composed of the gene expression profiles with Affymetrix microarray measured on the laboratory rat (Rattus norvegicus) cultured cells from SCN, which consists of the expression of 31,099 genes[11]. At the timepoint of 18h, the phase reset stimulus by drug forskolin was applied. The expressions of six genes (*Nr1d1*, *Arntl*, *Pfkm*, *RGD72*, *Per2* and *Cry1*), which are related to circadian rhythm, are predicted. We use the first $m = 16$ time points as the known series and make predictions for the next $L - 1 = 6$ time points.

Because the total 31,099 genes are not necessarily sharing the same attractor (biologically not all genes are in the same functional pathway), i.e., they are not necessarily intertwined. To make sure the one-to-one map exists, for each circadian rhythm-related gene, a set of $D = 84$ most related genes were selected to carry out the prediction. As shown in Fig. 5a of the main text, compared with other methods, ARNN works well (Table 1 of the main text) on the prediction of gene expressions, which demonstrates the effectiveness of ARNN in transforming high-dimensional spatial information to the temporal information of target genes.

6.7. Stock index dataset of the Shanghai Stock Exchange

The dataset of B-share Index of the Shanghai Stock Exchange is collected from totally $D = 1130$ stock indices (variables) with an interval of 1 day except Saturday and Sunday from 2018-05-01 to 2018-11-22, available via https://www.ricequant.com/doc/api/index/china. Each stock index is a relative number of stock price statistics that measure and reflect the overall price of the stock market and its changing trend. A representative stock index: the Shanghai Stock Exchange B-share Index was selected to predict. A series of $m = 50$ time points were set as the known series, while the future states at $L - 1 = 20$ time points were predicted. As shown in Fig. 5b and Table 1 of the main text, ARNN predicts the dynamic trends of one target index.

6.8. Hongkong cardiovascular inpatients dataset

This dataset contains several time series, including the indices series of air pollutants and the number series of cardiovascular inpatients in major hospitals in Hongkong[12]. Under the assumption that some of the cardiovascular inpatients were caused by the air pollutants, the ARNN was applied to forecast the inpatient numbers. Considering the delay effect of every potential factor as well as a dummy vector of weekday effect[13] we have a $D = 48$ dimensional system. A series of $m = 130$ time points were set as the known series, while the future states at $L - 1 = 60$ time points were predicted. Based on the daily concentrations of nitrogen dioxide (NO2), sulphur dioxide (SO2), ozone (O3), respirable suspended particulate (Rspar), mean daily temperature and relative humidity which were obtained from air monitoring stations in Hong Kong from 1994 to 1997, the ARNN method helps to predicted the short-term dynamical trend of the daily cardiovascular disease admissions (Fig. 5c of the main text).

6.9. Traffic speed dataset of Los Angeles County

This dataset contains the traffic speed collected from $D = 207$ loop detectors (variables) in the 134-highway of the Los Angeles County from 2012-03-01 to 2012-06-30[14]. ARNN uses the data at $m = 80$ time points, in four locations respectively and makes the predictions for the next $L - 1 = 30$ time points. As shown in Fig. 6 and Table 1 of the main text, ARNN predicts the traffic flow accurately. The movie of traffic speed is attached to the following link: https://github.com/RPcb/ARNN

6.10. MNIST dataset

To illustrate that the proposed framework is also capable in predicting spatial information, ARNN has also been applied to the handwriting digits 0-9 from the digit database MNIST. It is seen that the ARNN performs well in predicting the numbers (Supplementary Fig. 9). Each handwriting digit is originally demonstrated in 28×28 grids. In order to apply the ARNN, the spatial information of each image is converted to a 28-dimensional time series, that is, input an image column by column, with 28 being as the size of each input vector. For each prediction, the length of known pixels (input) is 5, while the length of predicting pixels is 2, i.e., 2 steps ahead. The satisfactory performance of ARNN shows the effectiveness of our method for the spatiotemporal pattern prediction.

Supplementary Note 7. Methods for comparison

In this study, we compared the performance of ARNN with that of the following methods.

7.1. Traditional Reservoir computing (tRC)

Traditional Reservoir Computing (tRC) is a unified computational framework[15,16], derived from independently proposed RNN models, such as echo state network (ESN)[17] and liquid state machine (LSM)[18]. Generally, ESN is the most studied RC framework.

ESN uses an RNN-based reservoir consisting of discrete-time artificial neurons. When the feedback from the output to the reservoir is absent, the time evolution of the neuronal states in the reservoir is described as follows[17]:

$$\mathbf{r}^t = f(W^{in}\mathbf{X}^t + W\mathbf{r}^{t-1}), \quad (11)$$

where t denotes the discrete time, $\mathbf{r}^t$ is the state vector of the reservoir units, $\mathbf{X}^t$ is the input vector, W^{in} is the weight matrix for the input-reservoir connections, and W is the weight matrix for the recurrent connections in the reservoir. In reservoir computing, both W^{in} and W are randomly given or fixed. The functions $f = (f_1, f_2, \dots, f_n)$ represents element-wise activation functions of the reservoir units, each of which is typically a sigmoid-type activation function. Supplementary Eq. (11) represents a non-autonomous dynamical system forced by the external input $\mathbf{X}^t$. The output is often given by a linear combination of the neuronal states as follows:

$$\mathbf{Y}^t = W^{out}\mathbf{r}^t, \quad (12)$$

where $\mathbf{Y}^t$ is the output vector and W^{out} is the weight matrix in the readout. In supervised learning, this weight matrix is trained to minimize the difference between the network output and the desired output for a certain time period. The performance of the ESN depends on the design of the RNN-based reservoir.

7.2. Autoregressive model (AR)

An autoregressive (AR) model is a time-series model that uses observations from previous time steps as input to a regression equation to predict the value at the next time step[19]. For example, calculating y^t from y^{t-1}:

$$y^t = \beta_0 + \beta_1 y^{t-1} + \epsilon^t. \quad (13)$$

In this regression model Supplementary Eq. (13), the response variable y^{t-1} in the previous time period has become the predictor and the errors ϵ^t have the usual assumptions about errors in a simple linear regression model. The order of an autoregression is the number of immediately preceding values in the series that are used to predict the value at the present time t. Here, Supplementary Eq. (13) is a first-order regression model. The multiple-order model used in the comparison is as the following Supplementary Eq. (14).

$$y^t = \beta_0 + \beta_1 y^{t-1} + \beta_2 y^{t-2} + \cdots + \epsilon^t. \quad (14)$$

7.3. Long short-term memory network (LSTM)

Long short-term memory network (LSTM) is an artificial recurrent neural network (RNN) architecture used in the field of deep learning[20]. A common LSTM unit is composed of a cell, an input gate, an output gate and a forget gate. The cell remembers values over arbitrary time intervals and the three gates regulate the flow of information into and out of the cell. An RNN using LSTM units can be trained in a supervised manner, on a set of training sequences, using an optimization algorithm, like gradient descent, combined with backpropagation through time to compute the gradients needed during the optimization process, in order to change each weight of the LSTM network in proportion to the derivative of the error (at the output layer of the LSTM network) with respect to corresponding weight.

7.4. Autoregressive integrated moving average (ARIMA)

AutoRegressive Integrated Moving Average (ARIMA) models[21] are typically expressed like "ARIMA (p, U, q)", with the three terms p, U, and q defined as follows:

- p means the number of preceding ("lagged") S values that have to be added/subtracted to S in the model, so as to make better predictions based on local periods of growth/decline in our data. This captures the "autoregressive" nature of ARIMA.
- U represents the number of times that the data have to be "differenced" to produce a stationary signal (i.e., a signal that has a constant mean over time). This captures the "integrated" nature of ARIMA.
- q represents the number of preceding/lagged values for the error term that are added/subtracted to S. This captures the "moving average" part of ARIMA.

7.5. Support vector regression (SVR)

Support vector regression (SVR) uses the supporting vector machine to fit curves and perform regression analysis[22]. It is a multi-variable method and the function used to predict new values is

$$f(x) = \sum_{n=1}^{N}(\alpha_n - \alpha_n^*)G(x_n, x) + b,$$

where x_n is a multivariable set of N observations with observed values x and $G(x_n, x) = e^{-|x_n - x|^2}$.

7.6. Radial basis function network (RBF)

A radial basis function network (RBF) is an artificial neural network that uses radial basis functions as activation functions[23]. The main feature of these functions is that their response decreases, or increases, monotonically with distance from a central point. The output of the network is a linear combination of radial basis functions of the inputs and neuron parameters.

7.7. Single-variable embedding (SVE)

Single Variable Embedding (SVE) is a forecast model which is based on the weighted average of the nearest neighbors in a single view[24]. The prediction is based on the trend of delay coordinates and only the time series of the target variable is used to make the predictions.

7.8. Multiview embedding (MVE)

Multiview embedding (MVE) examines the top *k* reconstructions, and uses the single nearest neighbor from each[25]. The MVE forecast (e.g., for variable *y*) is then defined as a simple average

$$\hat{y}_{t+1} = \frac{1}{k}\sum_{i=1}^{k} y_{nn^{i}(t)+1}$$

where $nn^i(t)$ is the time index of the nearest neighbor in the *i*-th attractor. MVE is intended to mitigate prediction errors that occur when nearest neighbors are misidentified or inaccurately weighted based on distance.

7.9. Linear method (Linear)

The linear method of the linearized STI equations (Eq. (2) of the main text) as follows

$$\begin{cases} A\mathbf{X}^t = \mathbf{Y}^t, \\ \mathbf{X}^t = B\mathbf{Y}^t, \end{cases}$$

where $AB = I$, A and B are $L \times D$ and $D \times L$ matrices, respectively, and I represents an $L \times L$ identity matrix. Clearly, the linearized STI equation is of the same structure of ARNN (Eq. (3) of the main text) except the reservoir/nonlinear part.

Supplementary references

1. Takens, F. Detecting strange attractors in turbulence. in *Dynamical systems and turbulence, Warwick 1980* 366–381 (Springer, 1981).
2. Sauer, T., Yorke, J. A. & Casdagli, M. "Embedology," Journal of Statistical Physics. (1991).
3. Ma, H., Zhou, T., Aihara, K. & Chen, L. Predicting time series from short-term high-dimensional data. *Int. J. Bifurc. Chaos* **24**, 1430033 (2014).
4. Ma, H., Leng, S., Aihara, K., Lin, W. & Chen, L. Randomly distributed embedding making short-term high-dimensional data predictable. *Proc. Natl. Acad. Sci.* **115**, E9994–E10002 (2018).
5. Chen, C. *et al.* Predicting future dynamics from short-term time series by anticipated learning machine. *Natl. Sci. Rev.* 7, 1079–1091 (2020).
6. Kingma, D. P. & Ba, J. Adam: A method for stochastic optimization. *ArXiv Prepr. ArXiv14126980* (2014).
7. Wang, H., Song, Y. & Tang, S. LSTM-based Flow Prediction. *ArXiv Prepr. ArXiv190803571* (2019).
8. Curry, J. H. A generalized Lorenz system. *Commun. Math. Phys.* **60**, 193–204 (1978).
9. Hirata, Y. & Aihara, K. Predicting ramps by integrating different sorts of information. *Eur. Phys. J. Spec. Top.* **225**, 513–525 (2016).
10. Zhang, K. & Fan, W. Forecasting skewed biased stochastic ozone days: analyses, solutions and beyond. *Knowl. Inf. Syst.* **14**, 299–326 (2008).
11. Wang, Y., Zhang, X.-S. & Chen, L. A network biology study on circadian rhythm by integrating various omics data. *OMICS J. Integr. Biol.* **13**, 313–324 (2009).
12. Wong, T. W. *et al.* Air pollution and hospital admissions for respiratory and cardiovascular diseases in Hong Kong. *Occup. Environ. Med.* **56**, 679–683 (1999).
13. Xia, Y. & Härdle, W. Semi-parametric estimation of partially linear single-index models. *J. Multivar. Anal.* **97**, 1162–1184 (2006).
14. Li, Y., Yu, R., Shahabi, C. & Liu, Y. Diffusion convolutional recurrent neural network: Data-driven traffic forecasting. *ArXiv Prepr. ArXiv170701926* (2017).
15. Verstraeten, D., Schrauwen, B., d'Haene, M. & Stroobandt, D. An experimental unification of reservoir computing methods. *Neural Netw.* **20**, 391–403 (2007).
16. Lukoševičius, M. & Jaeger, H. Reservoir computing approaches to recurrent neural network training. *Comput. Sci. Rev.* **3**, 127–149 (2009).

17. Jaeger, H. The “echo state” approach to analysing and training recurrent neural networks-with an erratum note. *Bonn Ger. Ger. Natl. Res. Cent. Inf. Technol. GMD Tech. Rep.* **148**, 13 (2001).

18. Maass, W., Natschläger, T. & Markram, H. Real-time computing without stable states: A new framework for neural computation based on perturbations. *Neural Comput.* **14**, 2531–2560 (2002).

19. Akaike, H. Autoregressive model fitting for control. in *Selected Papers of Hirotugu Akaike* 153–170 (Springer, 1998).

20. Hochreiter, S. & Schmidhuber, J. Long short-term memory. *Neural Comput.* **9**, 1735–1780 (1997).

21. Box, G. E. & Pierce, D. A. Distribution of residual autocorrelations in autoregressive-integrated moving average time series models. *J. Am. Stat. Assoc.* **65**, 1509–1526 (1970).

22. Kecman, V., Huang, T.-M. & Vogt, M. Iterative single data algorithm for training kernel machines from huge data sets: Theory and performance. in *Support vector machines: Theory and Applications* 255–274 (Springer, 2005).

23. Orr, M. J. *Introduction to radial basis function networks*. (Technical Report, center for cognitive science, University of Edinburgh, 1996).

24. Sugihara, G. & May, R. M. Nonlinear forecasting as a way of distinguishing chaos from measurement error in time series. *Nature* **344**, 734–741 (1990).

25. Ye, H. & Sugihara, G. Information leverage in interconnected ecosystems: Overcoming the curse of dimensionality. *Science* **353**, 922–925 (2016).
